# Feed-Forward Microprocessing and Splicing Activities at a MicroRNA–Containing Intron

**DOI:** 10.1371/journal.pgen.1002330

**Published:** 2011-10-20

**Authors:** Maja M. Janas, Mehdi Khaled, Steffen Schubert, Jacob G. Bernstein, David Golan, Rosa A. Veguilla, David E. Fisher, Noam Shomron, Carmit Levy, Carl D. Novina

**Affiliations:** 1 Department of Cancer Immunology and AIDS, Dana-Farber Cancer Institute, Department of Microbiology and Immunobiology, Harvard Medical School, Boston, Massachusetts, United States of America; 2 Broad Institute of Harvard and MIT, Cambridge, Massachusetts, United States of America; 3 Cutaneous Biology Research Center, Harvard Medical School, Charlestown, Massachusetts, United States of America; 4 MIT Media Lab, Massachusetts Institute of Technology, Cambridge, Massachusetts, United States of America; 5 Department of Cell and Developmental Biology, Sackler Faculty of Medicine, Tel Aviv University, Tel Aviv, Israel; 6 Department of Human Genetics and Biochemistry, Sackler Faculty of Medicine, Tel Aviv University, Tel Aviv, Israel; University of California San Francisco, United States of America

## Abstract

The majority of mammalian microRNA (miRNA) genes reside within introns of protein-encoding and non-coding genes, yet the mechanisms coordinating primary transcript processing into both mature miRNA and spliced mRNA are poorly understood. Analysis of melanoma invasion suppressor miR-211 expressed from intron 6 of melastatin revealed that microprocessing of miR-211 promotes splicing of the exon 6–exon 7 junction of melastatin by a mechanism requiring the RNase III activity of Drosha. Additionally, mutations in the 5′ splice site (5′SS), but not in the 3′SS, branch point, or polypyrimidine tract of intron 6 reduced miR-211 biogenesis and Drosha recruitment to intron 6, indicating that 5′SS recognition by the spliceosome promotes microprocessing of miR-211. Globally, knockdown of U1 splicing factors reduced intronic miRNA expression. Our data demonstrate novel mutually-cooperative microprocessing and splicing activities at an intronic miRNA locus and suggest that the initiation of spliceosome assembly may promote microprocessing of intronic miRNAs.

## Author Summary

MicroRNA (miRNA) genes are transcribed as long primary RNAs containing local hairpins that are excised by the Microprocessor complex minimally composed of Drosha and DGCR8. Most mammalian miRNAs reside in introns of protein-encoding and non-coding genes, but it is unclear how microprocessing of an intronic miRNA and splicing at the host gene intron affect each other. We recently reported that in melanoma, a miRNA expressed from intron 6 of melastatin (miR-211) assumes the tumor suppressive function of its host gene. In our current work, we detected elevated melastatin exon 6–exon 7 junctions relative to other exon-exon junctions that lack intronic miRNAs, suggesting that microprocessing promotes splicing. We show that microprocessing of miR-211 precedes completion of splicing of the exon 6–exon 7 junctions and that Drosha's endonuclease activity is required to facilitate exon 6–exon 7 junction formation. Additionally, we found that the first step of spliceosome assembly, recognition of the 5′ splice site by the U1 snRNP complex, promotes microprocessing of miR-211 and other intronic but not intergenic miRNAs. Our findings reveal a mutually cooperative, physical, and functional coupling of intronic miRNA biogenesis and splicing at the host intron, and they suggest a global positive effect of spliceosome assembly on intronic miRNA microprocessing.

## Introduction

Most eukaryotic primary transcripts undergo nuclear splicing, which removes introns and joins exons in a process catalyzed by a multi-megadalton complex called the spliceosome. Many protein-encoding and non-coding genes host small non-coding RNAs, among them hundreds of miRNAs [1]. In fact, most mammalian miRNAs are expressed from introns of protein-encoding and non-coding genes [2], [3]. MiRNA-containing hairpins are cropped from primary miRNA transcripts (pri-miRNAs) by the Microprocessor, a protein complex minimally containing the nuclear RNase III enzyme Drosha and DGCR8 [4]–[7]. Molecular mechanisms coordinating the activities of the spliceosome and the Microprocessor on primary transcripts generating both mature mRNAs and miRNAs are obscure.

Pri-miRNA processing may be physically coupled to transcription and/or splicing [8], [9], [10]. Pri-miRNA processing is more efficient if pri-miRNAs are retained at the transcription site [11] and clearance of introns following microprocessing of pri-miRNAs may enhance splicing efficiency [12], suggesting that microprocessing precedes the completion of splicing [2], [13]. Related, the processing of other classes of intronic small RNAs (such as snoRNAs) supports a model of cross-talk between small RNA processing and host gene splicing [14]. Additionally, splicing mutants in fission yeast reduce processing of centromeric transcripts into siRNAs and impair centromere silencing [15], suggesting that the spliceosome provides a platform that promotes siRNA biogenesis.

The melanocyte-specific gene melastatin and its hosted miR-211 gene located in intron 6 are robustly reduced in invasive human melanomas [16], [17]. Reconstitution of miR-211 but not melastatin suppressed melanoma invasion, implying distinct biological functions for these gene products expressed from a common primary transcript [16]. Surprisingly, we detected increased formation of exon 6-exon 7 junction relative to other melastatin exon-exon junctions which lack intronic miRNAs. Here we demonstrate that microprocessing of miR-211 promotes splicing of the exon 6-exon 7 junction of melastatin, that knockdown of Drosha and its binding partner DGCR8 reduces exon 6-exon 7 junction formation, and that the RNase III activity of Drosha is required to promote exon 6-exon 7 junction formation. We also report that splicing at intron 6 of melastatin promotes microprocessing of miR-211. Mutations in the 5′SS of intron 6 or knockdown of splicing factors interacting with the 5′SS reduced miR-211 biogenesis and Drosha recruitment to intron 6. Our analysis of miR-211 biogenesis from intron 6 of melastatin provides a mechanism for exon 6-exon 7 5′SS splice site recognition promoting miR-211 microprocessing, and miR-211 microprocessing promoting exon 6-exon 7 splicing.

## Results/Discussion

### Increased melastatin exon 6–exon 7 junction formation

**Figure 1:**
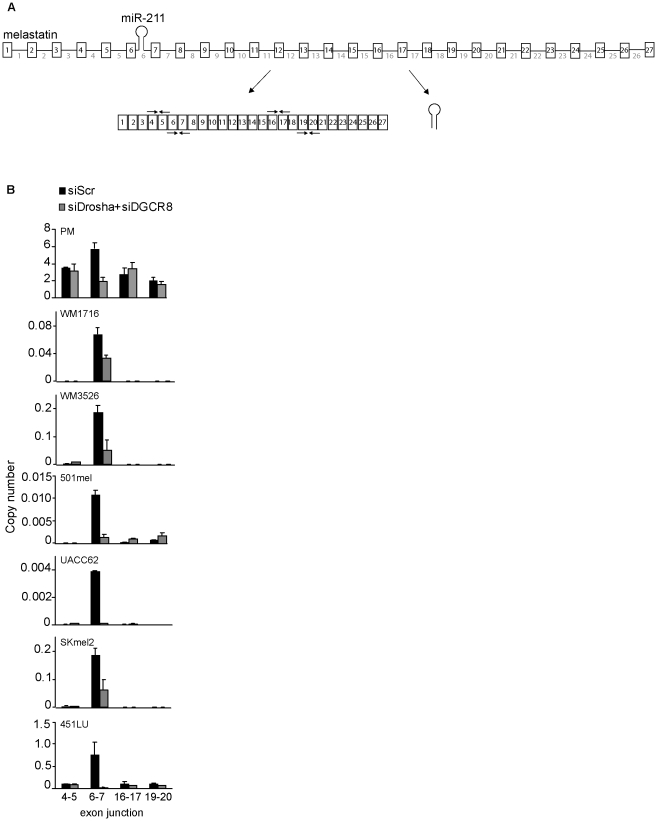
Microprocessor-dependent increases in melastatin exon 6–exon 7 junctions. (A) A schematic representation of melastatin primary transcript (consisting of 27 exons and 26 introns) producing both miR-211 from intron 6 and spliced melastatin mRNA. Arrow pairs represent qRT-PCR primers used to detect spliced exon-exon junctions. (B) miR-211-containing intron 6 of melastatin is preferentially spliced in primary melanocytes (PM), melanoma patient samples (WM1716 and WM3526), and melanoma cell lines (501mel, UACC62, SKmel2, and 451LU) in a Microprocessor-dependent manner. Relative copy numbers of indicated spliced exon-exon junctions, based on standard curves produced using cloned melastatin cDNA, were determined by qRT-PCR and normalized to Actin before and after Drosha and DGCR8 knockdowns.

To examine the effects of intronic miRNA microprocessing on host gene splicing in a biologically-relevant context, we compared spliced exon-exon junctions of miR-211 host gene melastatin (Figure 1A) in human primary melanocytes, human melanoma patient samples, and human melanoma cell lines (Figure 1B and Figure S1A). Consistent with previous reports [16], [17], [18], [19], melastatin was reduced in melanoma patient samples and melanoma cell lines compared to primary melanocytes. Surprisingly, we did not detect uniformly reduced exon-exon junctions across melastatin. In melanomas, splicing of the exons that flank the miR-211-containing intron 6 (exon 6-exon 7) was increased by 20–100 fold relative to other exon-exon junctions. The increased frequency of exon 6-exon 7 splicing was Microprocessor-dependent because knockdown of Microprocessor components Drosha and DGCR8 (Figure S1B) decreased exon 6-exon 7 junction formation by 2–100 fold but did not decrease (and in some cases increased) formation of other exon-exon junctions (Figure 1B). Consistent with these results, exon 6-intron 6 and intron 6-exon 7 junctions were selectively decreased relative to other exon-intron junctions of melastatin (Figure S1C), implying increased splicing efficiency at miR-211-containing intron 6. The Microprocessor-dependent two-fold increase in exon 6-exon 7 junctions relative to other exon-exon junctions was also observed in primary melanocytes (Figure 1B). We note that melastatin mRNA levels in primary melanocytes are 10–10,000 fold higher than in melanomas, suggesting that splicing of low-abundance primary transcripts is more sensitive to positive effects of hosted intronic miRNAs than splicing of high-abundance primary transcripts. The increased frequency of exon 6-exon 7 junctions likely was not due to an alternative transcription start site because neither upstream nor downstream exon-exon junctions were increased (Figure 1B and Figure S1A) and neither upstream nor downstream exon-intron junctions were decreased (Figure S1C), consistent with our previously-reported chromatin immunoprecipitation (IP) and micrococcal nuclease protection assays showing that melastatin and miR-211 are regulated by a common promoter [16]. These data suggest that microprocessing of miR-211 selectively increased the frequency of splicing at intron 6 of melastatin.

### Microprocessing of intronic miRNAs promotes splicing of the host introns

**Figure 2:**
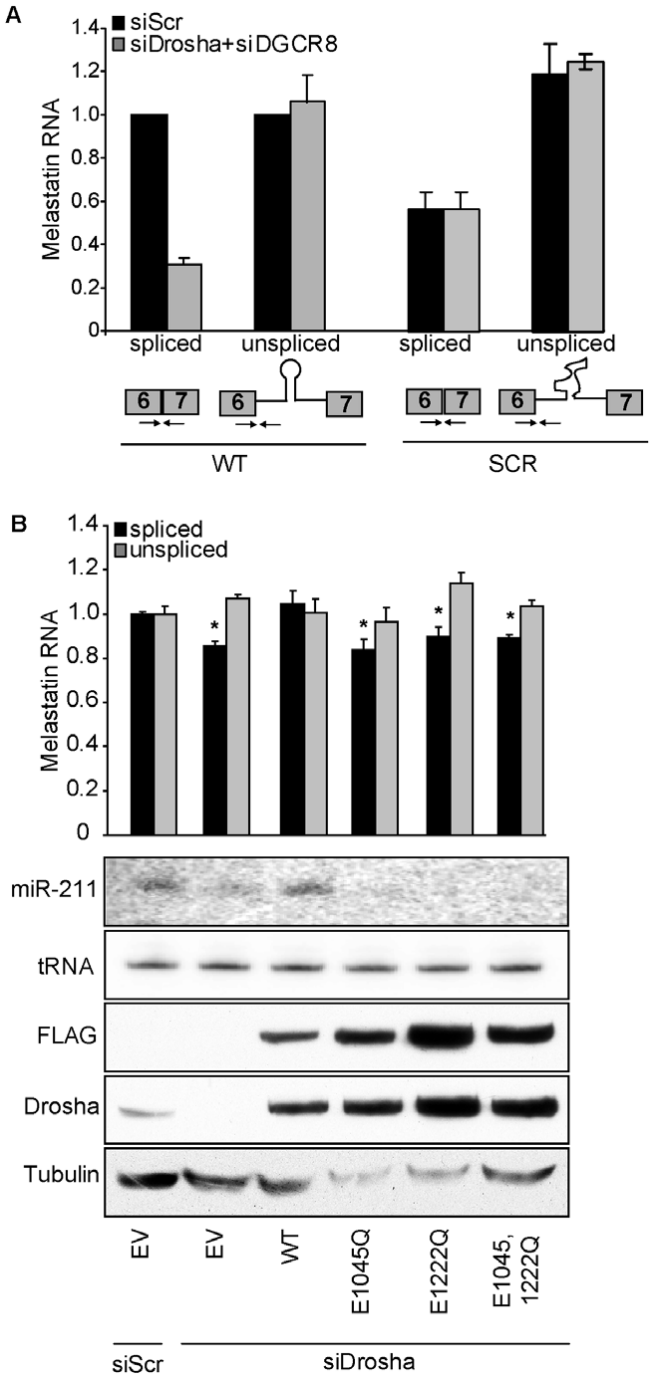
The RNase III activity of Drosha is required to promote splicing at a miR-211–hosting intron. (A) Eliminating the miR-211 hairpin from intron 6 reduces splicing efficiency of a melastatin mini-gene containing intron 6 flanked by exons 6 and 7. Wild-type (WT) mini-gene or a mini-gene in which the miR-211 hairpin was replaced by a scrambled sequence (SCR) were transfected into HeLa cells, and the levels of fully-spliced and unspliced transcripts were quantified by qRT-PCR before and after Drosha and DGCR8 knockdowns. (B) RNase III-inactive Drosha does not promote splicing of a miR-211-hosting intron. An empty vector (EV) or vectors containing siRNA-resistant isoforms of either WT or RNase III mutant (E1045Q, E1222Q, and E1045,1222Q) Drosha were co-transfected with WT melastatin mini-gene into HeLa cells treated with Scr or Drosha-specific siRNAs. Proteins (FLAG, Drosha, Tubulin) or RNAs (miR-211 and tRNA) were detected by Western and Northern blotting, respectively. Spliced and unspliced forms of the mini-gene transcript were quantified by qRT-PCR (p<0.05, Student's t-Test).

**Figure 3:**
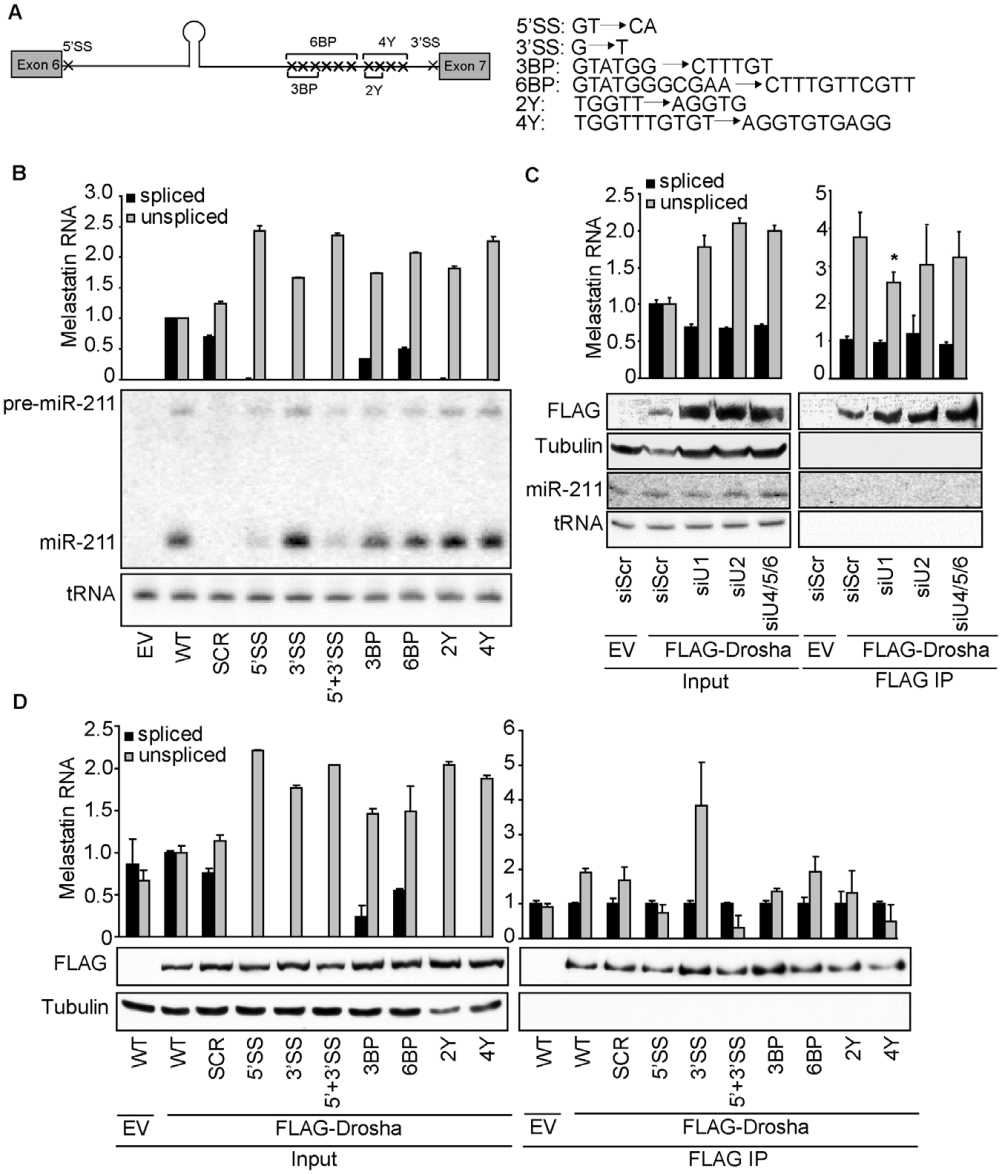
5′SS recognition promotes microprocessing of and Drosha recruitment to intronic miR-211. (A) WT melastatin mini-gene was mutated at indicated positions to generate constructs that lack the consensus 5′SS, the consensus 3′SS, three predicted branch points (3BP), six predicted branch points (6BP), two pyrimidines in the polypyrimidine tract (2Y), or four pyrimidines in the polypyrimidine tract (4Y). (B) Microprocessing of miR-211 is reduced by mutation of the 5′SS of the melastatin mini-gene, but is not affected by mutations in sequences involved in events downstream of 5′SS recognition. Either empty vector (EV) or vectors containing WT or mutant melastatin mini-genes were transfected into HeLa cells, and the efficiency of splicing was assessed by qRT-PCR and the efficiency of intronic miR-211 processing was assessed by Northern blotting. (C) Binding of Drosha to the miR-211-containing intron 6 of melastatin is reduced after depletion of splicing factors involved in U1 (SNRNP70+PRP8), but not U2 (U2AF65+PRP8) or U4/5/6 (PRP4+PRP8) snRNP function. HeLa cells were transfected with either Scr or splicing-factor-specific siRNAs, and then were co-transfected with vectors expressing WT melastatin mini-gene and either empty (EV) or FLAG-tagged Drosha. Anti-FLAG-Drosha immunoprecipitates and inputs were assayed for spliced and unspliced melastatin mini-gene transcripts by qRT-PCR (p<0.05). The efficiency of FLAG-Drosha immunoprecipitation was assessed by Western blotting. (D) Mutation of 5′SS of the melastatin mini-gene reduces Drosha binding to miR-211-hosting intron 6. Empty (EV) or FLAG-Drosha-expressing vectors were co-transfected into HeLa cells with indicated mini-gene vectors, and anti-FLAG-Drosha immunoprecipitates and inputs were analyzed by qRT-PCR for spliced and unspliced melastatin mini-gene transcripts. The efficiency of FLAG-Drosha immunoprecipitation was assessed by Western blotting (p<0.05, Student's t-Test).

To directly test whether microprocessing of miR-211 promoted splicing of melastatin exon 6-exon 7 junction, we constructed a melastatin mini-gene encompassing part of exon 6, entire intron 6, and part of exon 7, with either wild-type (WT) miR-211 or a scrambled sequence (SCR) that does not form an RNA hairpin (Figure 2A). We used HeLa cells for these experiments because HeLa cells express robust Microprocessor [5] and spliceosome [20] activities and do not express melastatin or miR-211 [21], [22], enabling precise control of experimental conditions. For all mini-gene vector transfections, we assessed the levels of miR-211 by qRT-PCR and Northern blotting, the levels of spliced mini-gene by exon 6-exon 7 qRT-PCR, the levels of unspliced mini-gene by exon 6-intron 6 qRT-PCR, and the steady-state mini-gene levels by exon 6 qRT-PCR (Table S1). To minimize detection of transfected plasmid DNA with primers intended for amplification of unspliced melastiatin mRNA, we treated RNA samples with DNaseI prior to RT reactions. We consistently detected four C_t_ value difference between +RT and the control –RT reactions, indicating that there was ∼16 fold less plasmid DNA than unspliced melastatin mRNA in our samples. Therefore, we do not believe that residual contaminating plasmid DNA influenced our quantitation of unspliced melastatin mRNA. Importantly, the primers used to detect spliced exon 6-exon 7 junctions gave no signal (C_t_ values ∼36–40) in –RT control reactions. To control for transfection efficiency, all mini-gene experiments were normalized to vector-expressed neomycin. Transfection of the WT mini-gene led to production of both pre-miR-211 and mature miR-211 as well as spliced exon 6-exon 7 junctions (Figure 2A, Figure S2A and Figure 3B). However, transfection of the SCR mini-gene abolished miR-211 production, reduced spliced exon 6-exon 7 junctions by two fold, and modestly increased unspliced exon 6-intron 6 junctions by up to 1.5 fold (Figure 2A, Figure S2A and Figure 3B). No difference was detected in the steady-state levels of mini-gene transcripts between the WT and SCR constructs (Figure S2A). Consistent with results in human melanocytes and melanomas, knockdown of Drosha and DGCR8 (Figure S2B) decreased WT mini-gene exon 6-exon 7 junction formation by three fold but did not affect SCR mini-gene exon 6-exon 7 junction formation (Figure 2A). Thus microprocessing of miR-211 from intron 6 promoted splicing of exon 6-exon 7 junctions of melastatin.

To rule out intron-specific effects of miR-211 on splicing, we cloned miR-211 or a SCR sequence into another melastatin mini-gene containing entire exon 20, entire intron 20, and entire exon 21 (Figure S2C). Consistent with intron 6 results, microprocessing of miR-211 from intron 20 increased exon 20-exon 21 splicing by 1.4 fold relative to the endogenous or SCR-containing mini-genes, suggesting that positive effects of miR-211 microprocesing on splicing are intronic context-independent. Next, to rule out miRNA-specific effects, we replaced miR-211 in intron 6 with another miRNA not expressed in HeLa cells, miR-124 (Figure S2D). Consistent with our results for the miR-211-expressing mini-gene, miR-124 microprocessing from intron 6 increased exon 6-exon 7 junctions by 1.4 fold and decreased unspliced exon 6-intron 6 junctions by 1.6 fold compared to the SCR construct. Together, these data show that increased splicing of at least two different host introns was dependent on the presence and microprocessing but not on the identity of a miRNA.

To distinguish whether binding or RNase III catalytic activity of Drosha promoted splicing of melastatin exon 6-exon 7, we knocked down endogenous Drosha in HeLa cells and tested the effects of ectopically-expressed siRNA-resistant WT or RNase III mutant Drosha on melastatin mini-gene splicing (Figure 2B). In these experiments, Drosha knockdown decreased WT mini-gene splicing by up to 1.3 fold. These smaller effects (compared to a three-fold decrease in Figure 2A) might be due to the absence of DGCR8 knockdown and/or the timing of the reconstitution experiments, in which endogenous Drosha was knocked down for 48 hrs before reconstitution (instead of 72 hrs as in Figure 2A). Rescue of endogenous Drosha knockdown with ectopic WT Drosha restored miR-211 microprocessing and exon 6-exon 7 junction formation. In contrast, ectopic expression of Drosha RNase III mutants (E1045Q, which abolishes endonuclease activity at 3′ strands of miRNA hairpins; E1222Q, which abolishes endonuclease activity at 5′ strands of miRNA hairpins [6]; or combined E1045,1222Q) failed to restore miR-211 microprocessing from the melastatin mini-gene and also failed to rescue decreased exon 6-exon 7 junction formation after endogenous Drosha knockdown. Importantly, these RNase III mutants do not affect the pri-miRNA binding activity of Drosha [6]. These results demonstrate the requirement for Drosha RNase III activity to promote splicing at the miR-211-containing intron 6 of melastatin. Because abolishing Drosha endonuclease activity at either 5′ or 3′ strands of miRNA hairpins failed to promote exon 6-exon 7 junction formation, our data imply that completion microprocessing precedes completion of splicing, consistent with previous reports [2], [13].

### 5′SS recognition promotes microprocessing of miR-211

Positive and negative effects of spliceosome-interacting proteins on miRNA biogenesis suggest that primary transcript splicing may affect microprocessing of hosted intronic miRNAs [8], [9]. For instance, the KH-type splicing regulatory protein (KSRP) is an AU-rich element binding protein [23] that interacts with Drosha to promote biogenesis of a subset of miRNAs by binding to G-rich stretches in terminal loops of miRNA precursors [24], [25]. Additionally, heterogeneous nuclear ribonucleoprotein A1 (hnRNP A1), which binds to nascent transcripts and couples transcription and splicing with mRNA export, has been shown to antagonize KSRP-mediated biogenesis of certain miRNAs [26] and promote biogenesis of other miRNAs [27]. The terminal loop of miR-211 possesses a G-rich stretch and intronic sequences surrounding miR-211 possess AU-rich elements, suggesting that splicing might affect miR-211 microprocessing by recruitment of KSRP or hnRNP A1.

To directly test the effects of splicing on intronic miRNA biogenesis, we introduced point mutations in the consensus sequences of the 5′SS, 3′SS, branch points, or polypyrimidine tract of the miR-211-containing melastatin mini-gene (Figure 3A). When transfected into HeLa cells, these mutations reduced fully-spliced mini-gene RNA levels by 2–100 fold and increased unspliced mini-gene RNA levels by up to 2.5 fold (Figure 3B). Interestingly, only mutations in the 5′SS reduced the levels of pre-miR-211 and mature miR-211. In contrast, mutations in the 3′SS or polypyrimidine tract modestly increased miR-211 levels, while branch point mutations had no effect on miR-211 levels. Neither miR-211 nor any mini-gene sequences were detected in HeLa cells transfected with an empty control vector, as expected. Thus 5′SS recognition facilitates miR-211 microprocessing from intron 6 of melastatin, while 3′SS recognition may have a slight inhibitory effect.

To rule out cell line-specific effects, we also transfected WT and mutant mini-gene constructs into two other human cancer cell lines, kidney HEK293T and lung A549 (Figure S2E). Consistent with data form HeLa cells, miR-211 microprocessing from constructs containing 5′SS mutation (5′SS and 5′+3′SS mutants) was strongly reduced in both cell lines, as assessed by Northern blotting for pre-miR-211 and miR-211. Also in agreement with HeLa experiments, we observed decreased spliced exon 6-exon 7 junction formation when miR-211 was replaced by a SCR sequence, as assessed by qRT-PCR. These data suggest that the cooperativity between splicing and microprocessing is cell type-independent. Next, to rule out miRNA-specific effects of splicing on microprocessing, we introduced 5′SS, 3′SS, or 5′+3′SS mutations into the mini-gene containing miR-124 in intron 6 (Figure S2D). Consistent with miR-211 results, miR-124 microprocessing was significantly reduced in constructs containing 5′SS mutation. However, in contrast to miR-211 results, 3′SS mutation reduced miR-124 microprocessing to the same degree as 5′SS mutation, and combined 5′+3′SS mutation abolished miR-124 microprocessing. These data suggest that 5′SS recognition complex binding promotes microprocessing of intronic miRNAs in a miRNA-independent manner, and that the effects of the 3′SS recognition complex on microprocessing are miRNA-dependent. Thus, the molecular mechanisms of positive effects of splicing on microprocessing may be miRNA-dependent.

To confirm that decreased miR-211 biogenesis after 5′SS mutation was due to reduced spliceosome activity rather than to an artifact of mini-gene sequence alteration, we knocked down splicing factors that function in different steps of spliceosome assembly. Because the spliceosome is a dynamic multi-megadalton complex which exhibits redundancy [28], knockdown of individual splicing factors did not significantly affect splicing of the miR-211-containing melastatin mini-gene (data not shown). We therefore knocked down the central splicing factor PRP8 in combination with either a splicing factor unique to U1 (SNRNP70) which binds 5′SS, U2 (U2AF65) which binds the branch point, polypyrimidine tract and 3′SS, or U4/5/6 (PRP4) which bridges U1 and U2 and eventually rearranges the spliceosome for catalysis of exon joining and lariat intron release (reviewed in [28]). Knockdown of U1-, U2- and U4/5/6-specific factors (Figure S3A) increased the levels of the unspliced mini-gene by up to two fold and decreased the levels of the spliced mini-gene by 1.6 fold (Figure 3C). Importantly, steady-state mini-gene levels were not altered in the knockdowns (Figure S3B). Consistent with our mutational analysis of the melastatin mini-gene splice sites, only knockdown of SNRNP70, but not U2AF65 or PRP4, reduced miR-211 biogenesis from the WT mini-gene by up to 1.5 fold (Figure 3C). The reduction in miR-211 levels upon knockdown of 5′SS interacting factors is smaller compared to the reduction in miR-211 levels after 5′SS mutation likely because 5′SS mutation completely abolished splicing (Figure 3B) while SNRNP70 and PRP8 knockdown decreased splicing only by 1.6 fold, indicating incomplete depletion and functional redundancy. These data further demonstrate that 5′SS recognition by U1 precedes and promotes microprocessing of miR-211 and that microprocessing and splicing of miR-211 are mechanistically-coupled processes.

To test whether microprocessing and splicing of miR-211 are coupled through direct protein-protein interaction or secondarily through simultaneous interaction with a common primary transcript, we performed co-IP analyses in the presence and absence of RNase A (Figure S3C). Transfection of FLAG-Drosha followed by anti-FLAG IP identified association of U1, U2, U4, U5, and U6 snRNAs even at high concentrations (60 ng/mL) of RNaseA. These data demonstrate that Drosha can directly interact with the spliceosome independently of contacts with primary transcripts, as suggested previously [5], [9], [13]. Therefore, one possible explanation for reduced microprocessing of intronic miR-211 from the melastatin mini-gene after perturbation of 5′SS recognition (either by the 5′SS mutation or knockdown of U1-specific SNRNP70) is that Drosha interaction with intronic miR-211 is stabilized by the spliceosome complex formed at the 5′SS.

To assess whether perturbation of the spliceosome assembly at the 5′SS affects Drosha binding to miR-211-containing intron, we analyzed the association of WT and mutant melastatin mini-genes with Drosha (Figure 3D). Anti-FLAG-Drosha IPs were assessed by qRT-PCR for spliced and unspliced mini-gene RNA. We calculated IP efficiency using the formula: (mini-gene_IP_/GAPDH_IP_)/(mini-gene_INPUT_/GAPDH_INPUT_). Thus, a value of one indicates no enrichment of that RNA in Drosha IP despite background detection of both mini-gene RNA and GAPDH in IP, which we minimized through extensive washing and gentle elution with FLAG peptide. Consistent with decreased miR-211 microprocessing from 5′SS mutant mini-gene, 5′SS mutation decreased Drosha association with the unspliced mini-gene RNA by two fold. Also consistent with modestly increased miR-211 microprocessing from 3′SS mutant mini-gene, 3′SS mutation increased Drosha association with the unspliced mini-gene RNA by three fold. Additionally, only knockdown of SNRNP70, but not U2AF65 or PRP4, significantly reduced the association of the WT mini-gene with Drosha by 1.5 fold as assessed by anti-FLAG immunoprecipitation after splicing factor depletions (Figure 3C). As expected, no enrichment of the spliced mini-gene RNA in anti-FLAG-Drosha immunoprecipitates was observed for all mini-gene constructs. Thus, the 5′SS recognition complex assembly promotes the association of Drosha with miR-211-containing intron 6 of melastatin, increasing microprocessing.

A previously-proposed model suggested that splicing and microprocessing of intronic miRNAs were functionally-independent processes [2]. These studies demonstrated that intronic miRNAs can be processed from unspliced introns in cells and that microprocesing occurs before splicing completion in vitro. These studies also showed that the presence of an intronic miRNA did not affect (and in some cases modestly decreased) splicing efficiency, while spliceosome assembly modestly increased microprocessing. Our data is consistent with the model of microprocessing preceding splicing completion. Specifically, we demonstrate that U1 recognition of the 5′SS precedes and promotes Drosha binding to and microprocessing of miR-211 or miR-124, which precedes and promotes completion of splicing in an intronic context-independent manner. Moreover, we identified novel, interdependent, mutually-cooperative Microprocessor and spliceosome activities at the miR-211 locus that are directly coupled through protein-protein interactions [2], [13]. Consistent with a positive effect of splicing on microprocessing, introducing a miRNA hairpin within a synthetic intron improves the silencing efficiency of RNAi vectors [29]. It is possible that sequence determinants (e.g. miRNA hairpin loop, miRNA hairpin flanking sequences, exonic or intronic splicing enhancers and silencers, or other contextual parameters) affects coupling between microprocessing and splicing of intronic miRNAs.

### Knockdown of U1 splicing factors reduces intronic miRNAs globally

**Figure 4:**
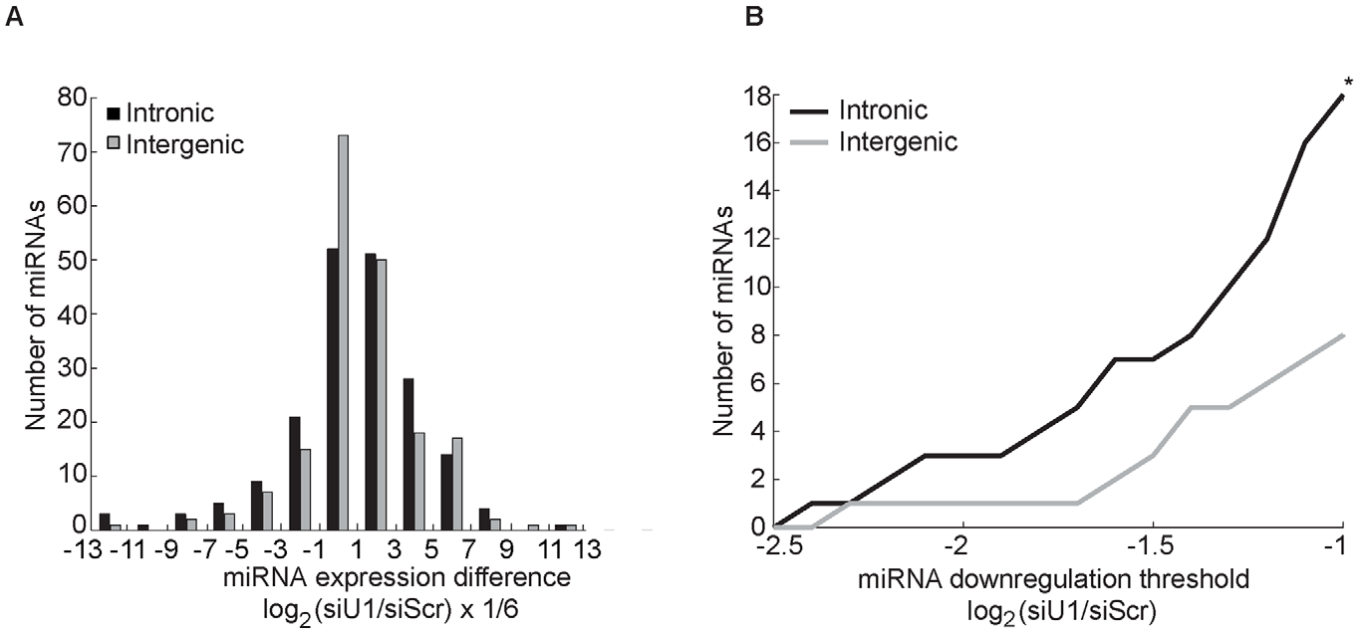
Knockdown of factors in the 5′SS recognition complex U1 globally decreases intronic miRNA levels. (A) Human melanoma cell lines, 451LU and 501mel, were transfected with either Scr or U1-specific (SNRNP70+PRP8) siRNAs, and total RNA was extracted and subjected to miRNA expression profiling. The effect of U1 depletion on 192 intronic (black bars) and 190 intergenic (gray bars) miRNAs detected in either cell line is represented as a histogram with the number of miRNAs on the y-axis plotted as a function of the normalized log_2_ difference in the levels between control and U1 depletion on the x-axis. The x-axis bins sizes are 1/3 of a unit; the bins are centered at log_2_ fold change of 0, +−1/3, +−2/3,…. (B) Total number of intronic and intergenic miRNAs (y-axis) graphed as a function of fold down-regulation (x-axis). 18 intronic and 8 intergenic miRNAs were down-regulated by more than 2 fold (log_2_(siU1/siScr)<−1; p<0.05).

The only evolutionarily-conserved portion of intron 6 of melastatin corresponds to the miR-211 hairpin, arguing against the presence of conserved regulatory sites in this intron. Still, cryptic or unknown regulatory elements may be present in intron 6 or intron 20 and thus our observations may be a unique to miR-211 and melastatin. To test whether the effects of splicing on miR-211 and miR-124 microprocessing can be generalized to other intronic miRNAs, we knocked down the 5′SS recognition factor SNRNP70 with PRP8 in two human melanoma cell lines (451LU and 501mel) and performed miRNA microarray. Of the 192 intronic and 190 intergenic miRNAs detected (Figure 4A and Table S2), 18 intronic but only six intergenic miRNAs were down-regulated by more than two fold after U1 knockdown (p<0.05; Figure 4B and Table S3). Reduced levels of these intronic miRNAs were independently validated by qRT-PCR (Figure S4). As expected, intronic miR-211 levels decreased after U1 knockdown by 1.2 fold in both melanoma cell lines (Table S2 and Figure S4). For all miRNA/miRNA* pairs detected in the most highly down-regulated group (four intronic miRNAs and four intergenic miRNAs), when the miRNA strand was reduced by more than two fold, the miRNA* strand was also reduced (Table S3). Similarly, when the miRNA* strand was reduced by more than two fold, the miRNA strand was also reduced, supporting miRNA duplex biogenesis defect upon U1 depletion. Importantly, the majority of intronic miRNAs that were reduced by more than two fold in one cell line were also reduced (by less than two fold) in the other cell line (Table S3). In contrast, the majority of intergenic miRNAs that were reduced by more than two fold in one cell line were increased in the other cell line, indicating a universal mechanism for the U1 splicing complex promoting biogenesis of intronic but not intergenic miRNAs. Thus intronic miRNAs were preferentially reduced upon U1 depletion relative to intergenic miRNAs. These findings suggest that 5′SS recognition complex may globally promote microprocessing of intronic miRNAs, consistent with out detailed analyses of miR-211 and miR-124 microprocessing from intron 6 of melastatin.

**Figure 5:**
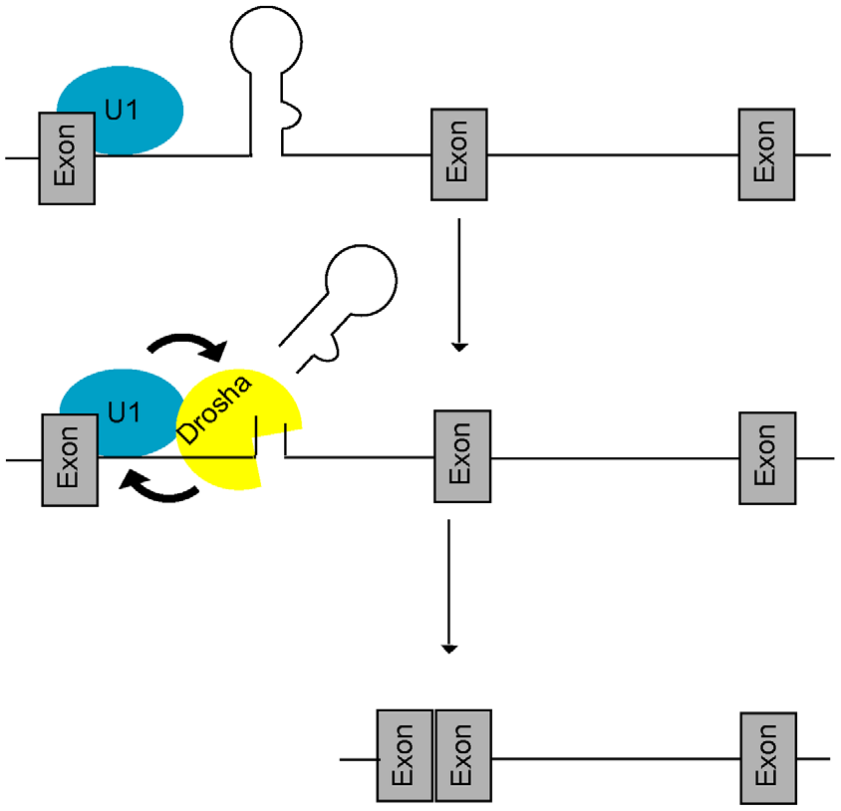
A feed-forward model of microprocessing and splicing. At intronic miRNA loci, the U1 complex first recognizes the 5′SS and promotes (arrow) recruitment of Drosha, leading to increased microprocessing and rapid clearance of the miRNA hairpin. Increased cropping by Drosha in turn generates a better splicing substrate by stabilizing both the cut intron and 5′SS binding by the U1 complex (arrow) thus promoting splicing completion. Mutually-cooperative splicing and microprocessing activities result in increased exon-exon junction formation at intronic miRNA loci.

Here we demonstrate a feed-forward loop between microprocessing and splicing, whereby 5′SS recognition by the U1 complex promotes microprocessing of intronic miR-211 by Drosha (possibly through recruitment of factors that promote microprocessing, such as KSRP and hnRNP A1), and microprocessing of miR-211 promotes splicing at its host melastatin intron 6 (Figure 5). Disruption of 5′SS recognition both in cis (mutations of 5′SS splice site) and in trans (knockdown of U1 splicing factors) decreased processing of miR-211 and, conversely, inhibition of the Microprocessor activity reduced splicing of intron 6 of melastatin. Because RNase III-deficient Drosha was unable to promote exon 6-exon 7 junction formation, our model implies that rapid Microprocessor cropping promotes splicing, possibly by enabling intronic RNA degradation in preparation for splicing, as suggested previously [12].

We note that the biogenesis of mirtrons [30], [31] is mechanistically distinct from the class of intronic miRNAs described here. Mirtrons are expressed from very short introns in which splicing substitutes for microprocessing and thus mutually-cooperative activities between splicing and microprocessing do not exist. It is notable that the debranched intron lariat possesses a phosphorylated 5′ end that is recessed relative to the overhanging 3′ hydroxylated end [30], [31], enabling mirtron recognition by exportin 5 and participation in cytoplasmic miRNA pathways. At one level, therefore, splicing appears to have co-evolved with microprocessing, at least in the case of the mirtron class of intronic miRNAs.

## Materials and Methods

### Cell culture, RNA extraction, and qRT–PCR

Primary melanocytes, melanoma patient samples, melanoma cell lines, HeLa, HEK293T, and A549 cells were cultured in DMEM supplemented with 10% fetal bovine serum and 1% Penicillin-Streptomycin-Glutamine (Invitrogen). Total RNA was extracted with Trizol (Invitrogen) according to manufacturer's instructions. For qRT-PCR analysis of miRNAs and RNU58b, 10 ng total RNA was treated with RNase-free DNase (Qiagen), reverse-transcribed and quantified with TaqMan microRNA assay kit with supplied primers (Applied Biosystems), according to manufacturer's instructions. For qRT-PCR analysis of the melastatin mini-gene cassette, 100 ng of total RNA was treated RNase-free DNase (Qiagen), reverse-transcribed using Quantitect kit (Qiagen), and quantified using iQ SYBR-Green Supermix (Biorad). MiRNA expression profiling was performed using TaqMan Low Density Array (Applied Biosystems).

### Oligonucleotide transfection

SiRNAs were transfected using HiPerFect (Qiagen) according to manufacturer's instructions. Vectors were transfected using Lipofectamine2000 (Invitrogen) according to manufacturer's instructions. All siRNAs were purchased from Ambion and had the following target sequences: PRP8 (5′ CCCUACAUGUGAACAACGATT 3′); U2AF65 (5′ CCAACUACCUGAACGAUGATT 3′); SNRNP70 (5′ GGUCUACAGUAAGCGGUCATT 3′); Drosha (5′ GACCAGACUUUGUACCCUUTT 3′); DGCR8 (5′ GGAUCAUGACAUUCCAUAATT 3′); PRP4 (5′ UCAUGGCGCUUAUGGGAUU 3′); Scr control siRNA (Ambion).

### Construction of the melastatin mini-gene vector and mutagenesis

A fragment of melastatin containing the 3′ end of exon 6, entire intron 6, and the 5′ end of exon 7 was amplified from the BAC clone RP11-348B17 (Children's Hospital Oakland Research Institute). Sizes: intron-2783 nt; exon 6–151 nt (full-size-172 nt); exon 7–108 nt (full-size-175 nt); pre-miRNA-110 nt; intron upstream of pre-miRNA-934 nt; intron downstream of pre-miRNA-1739 nt. The fragment was digested with HindIII and BamHI restriction enzymes, and inserted into pcDNA3.1 vector (Invitrogen). Site directed mutagenesis was performed using the quick change method from Stratagene according to the manufacturer's protocols. miR-211 was replaced by miR-124 in intron 6 by introducing AgeI and SacII or AgeI and PmlI restriction sites around miR-211, and ligating pre-miR-124 using annealed DNA oligos (IDT) with AgeI and SacII or PmlI overhangs. A fragment of melastatin containing entire exon 20, entire intron 20, and entire exon 21 was amplified from the BAC clone RP11-348B17 (Children's Hospital Oakland Research Institute). AgeI and EcoRI restriction sites were introduced in intron 20, and either SCR or pre-miR-211 sequences were ligated using annealed DNA oligos (IDT) with AgeI and EcoRI overhangs.

### Northern blot analysis

Five micrograms of total RNA were resolved on a 12% Urea–Polyacrylamide gel (BioRad) and transferred to a Hybond-N+ membrane (Amersham). The membrane was dried, UV crosslinked, pre-incubated with ULTRAhyb-Oligo Hybridization Buffer (Ambion) for 1 h, and incubated overnight at 42°C with an antisense probe directed against mature miR-211, miR-29a, or tRNA (miR-211 probe: 5′ rArGrGrCrGrArArGrGrArUrGrArCrArArArGrGrGrArA 3′; miR-29a probe: 5′ rArArCrCrGrArUrUrUrCrArGrArUrGrGrUrGrCrUrArG 3′; tRNA DNA probe: 5′ TGGTGGCCCGTACGGGGATCGA 3′). Probes were 5′ end-labeled with PNK (New England Biolabs) or using mirVana Probe and Marker kit (Ambion). The membrane was washed for 10 min at 42°C in 2× SSC, 0.1% SDS, and for 10 min at 42°C in 0.2× SSC, 0.1% SDS, exposed and scanned using a Storm PhosphorImaging system (Molecular Dynamics). Sizes of mature miRNAs were confirmed using a labeled small RNA ladder (Ambion).

### Antibodies

The following antibodies were used: Drosha 07-717 (Upstate), Actin 13E5 #4970 (Cell Signaling), FLAG 2368 (Cell Signaling), and GAPDH 2118 (Cell Signaling).

### Immunoprecipitation

HeLa cells were lysed in RIPA buffer containing 10 mM Tris pH 8.0, 150 mM NaCl, 1% Triton X-100, 0.5% sodium deoxycholate, 0.1% SDS, 1 mM EDTA, 0.5 mM EGTA, 0.4 U/uL RNase inhibitors, and a protease inhibitor cocktail tablet, and centrifuged at 12,000 g for 15 min at 4°C. Mouse IgG agarose (Sigma) and anti-FLAG M2 agarose (Sigma) were washed in RIPA. After pre-clearing lysates for 1 hr at 4°C with mouse IgG agarose, IP was performed for 1 hr at 4°C with anti-FLAG agarose pre-blocked with BSA and tRNA. After washing the beads five times with RIPA, complexes were eluted with 150 ug/ml FLAG peptide in lysis buffer by shaking for 30 min at 4°C.

## Supporting Information

Figure S1 Endogenous miR-211-containing intron 6 of melastatin is preferentially spliced in a Microprocessor-dependent manner. (A) Relative copy numbers of indicated exon-exon junctions, based on standard curves produced using cloned melastatin cDNA, were determined by qRT-PCR and normalized to Actin in primary melanocytes (PM) and melanoma cell lines (UACC62, SKmel2, and 501mel). (B) Knockdown efficiencies of Drosha and DGCR8 in melanomas and primary melanocytes (PM) were assessed by qRT-PCR and normalized to Actin. (C) Relative expression levels of indicated exon-intron junctions across melastatin primary transcript in melanoma cell line 501mel were assessed by qRT-PCR and normalized to Actin.
(TIF)

Figure S2 Cooperativity between splicing and microprocessing is miRNA- and intronic context-independent. (A) Replacing miR-211 with a SCR sequence in the melastatin mini-gene decreases miR-211 expression and exon 6-exon 7 splicing, but not steady-state mini-gene levels. WT or SCR melastatin mini-gene were transfected into HeLa cells, and the levels of miRNAs (intronic miR-211 and intergenic miR-29a) and mini-gene transcripts (using primers that specifically amplify exon 6, exon 6-exon 7, and pre-miR-211) were assessed by qRT-PCR. (B) Knockdown efficiencies of Drosha and DGCR8 in HeLa cells were assessed by qRT-PCR and normalized to Actin. The functionality of knockdowns was confirmed by qRT-PCR for miR-29a and miR-211. (C) miR-211 microprocessing promotes splicing in an intron-independent manner. miR-211 or a SCR sequence were cloned intro a second melastatin mini-gene containing entire exon 20, entire intron 20, and entire exon 21. HeLa cells were transfected with an empty vector (EV), miR-211-containing mini-gene (+miR-211), SCR-containing mini-gene (+SCR), or completely endogenous mini-gene (−). miR-211 expression was assessed by Northern blotting normalized to tRNA, and exon 20-exon 21 splicing was assessed by qRT-PCR normalized to neomycin. (D) Positive effects of 5′SS recognition on microprocessing and microprocessing on splicing are miRNA-independent. miR-211 in the exon 6-intron 6-exon 7 melastatin mini-gene was replaced by miR-124, and the effects of miR-124 microprocessing on splicing and splicing on miR-124 microprocessing were assessed after transfection of WT and mutant mini-genes into HeLa cells. Spliced exon 6-exon 7 junctions and unspliced exon 6-intron 6 junctions were assessed by qRT-PCR normalized to neomycin, and miR-124 expression was assessed by Northern blotting normalized to tRNA. (E) Positive effects of 5′SS recognition on microprocessing and microprocessing on splicing are cell type-independent. Either empty vector (EV) or vectors containing WT or mutant melastatin mini-genes were transfected into HEK293T and A549 cells, and the efficiency of splicing was assessed by qRT-PCR and the efficiency of intronic miR-211 processing was assessed by Northern blotting.
(TIF)

Figure S3 The spliceosome and the Microprocessor are mechanistically and physically coupled. (A) Knockdown efficiencies of indicated splicing factors (PRP8, U2AF65, and SNRNP70) in HeLa cells were assessed by qRT-PCR and normalized to Actin. (B) Knockdown of U1 (SNRPNP70+PRP8) and U2 (U2AF65+PRP8) splicing factors decreases exon 6-exon 7 splicing but not mini-gene transcript steady-state levels. Scr or splicing factor-specific siRNAs were transfected into HeLa cells, and the levels of mini-gene transcripts (using primers that specifically amplify exon 6, exon 6-exon 7, and pre-miR-211) were assessed by qRT-PCR. (C) The interaction between Drosha and the spliceosome is RNA-independent. Empty (−) or FLAG-Drosha-expressing (+) vectors were transfected into HeLa cells, and FLAG-Drosha was immunoprecipitated with anti-FLAG beads in the absence or presence of increasing concentrations of RNaseA. Inputs and anti-FLAG-Drosha immunoprecipitates were analyzed for proteins (FLAG-Drosha and GAPDH) and RNAs (U1, U2, U4, U5A, U6, and tRNA) by Western and Northern blotting, respectively.
(TIF)

Figure S4 Validation of miRNA expression profiling data. Intronic miRNAs that decreased more than two-fold after U1 (SNRNP70+PRP8) knockdown as assessed by the microarray were validated by qRT-PCR and normalized to U48 in the indicated melanoma cell lines.
(TIF)

Table S1 Primers used in these studies.
(DOC)

Table S2 Normalized miRNA expression levels (Ct value threshold ≤30) after U1 (SNRNP70+PRP8) knockdown in melanoma cell lines 451LU and 501mel as detected by the microarray.
(DOC)

Table S3 Intronic and intergenic miRNAs that decrease at least two fold after U1 (SNRNP70+PRP8) knockdown in melanoma cell lines 451LU and 501mel as detected by the microarray; #, miRNA* strand; N/A, not detected by the microarray.
(DOC)
