# Supplementary figures and images for: Feed-Forward Microprocessing and Splicing Activities at a MicroRNA–Containing Intron
Source: PLoS Genet. 2011 Oct 20;7(10):e1002330. doi: 10.1371/journal.pgen.1002330 (PMC3197686; doi:10.1371/journal.pgen.1002330)

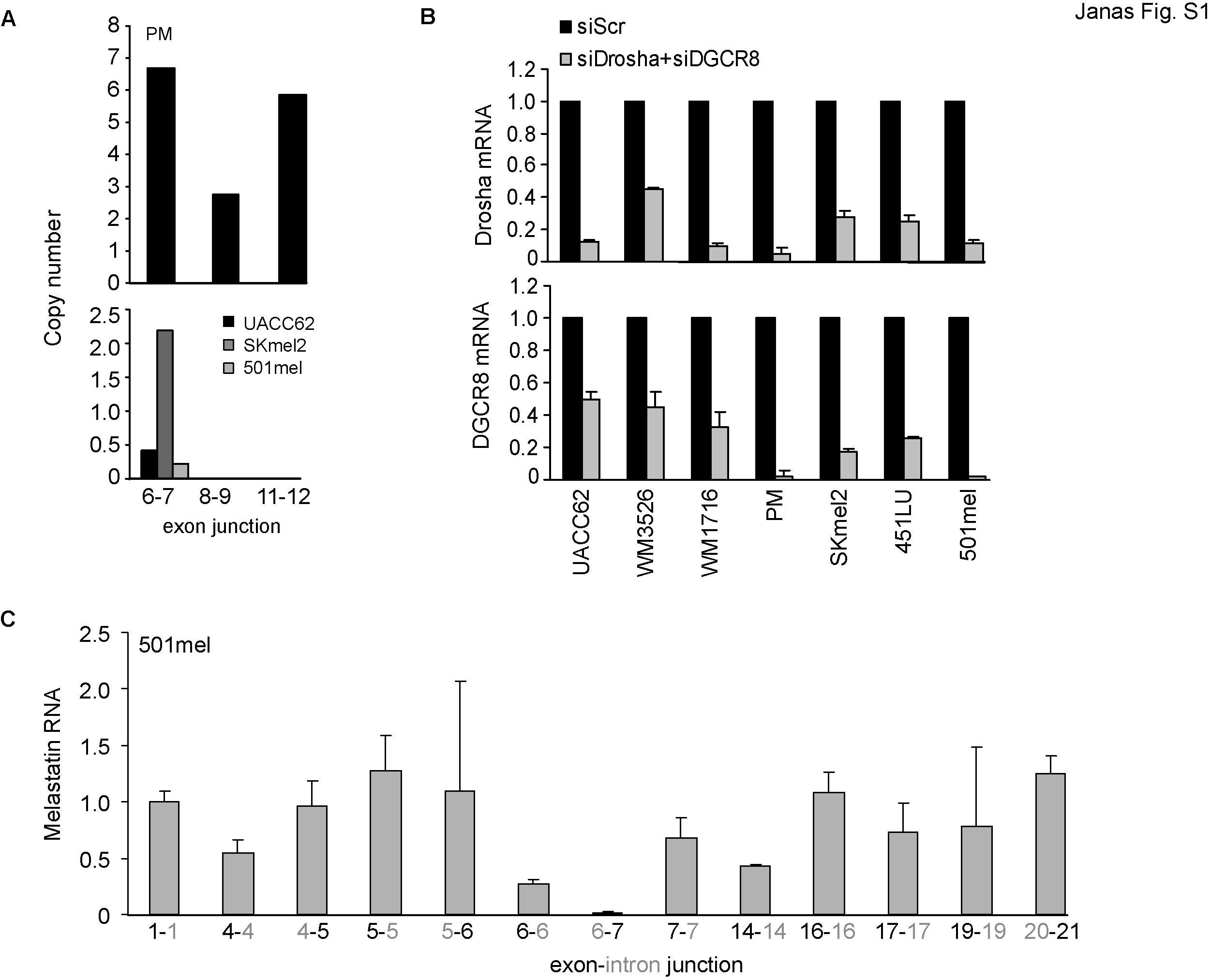

Supplement: Figure S1 — Endogenous miR-211-containing intron 6 of melastatin is preferentially spliced in a Microprocessor-dependent manner. (A) Relative copy numbers of indicated exon-exon junctions, based on standard curves produced using cloned melastatin cDNA, were determined by qRT-PCR and normalized to Actin in primary melanocytes (PM) and melanoma cell lines (UACC62, SKmel2, and 501mel). (B) Knockdown efficiencies of Drosha and DGCR8 in melanomas and primary melanocytes (PM) were assessed by qRT-PCR and normalized to Actin. (C) Relative expression levels of indicated exon-intron junctions across melastatin primary transcript in melanoma cell line 501mel were assessed by qRT-PCR and normalized to Actin. (TIF) [file pgen.1002330.s001.tif]

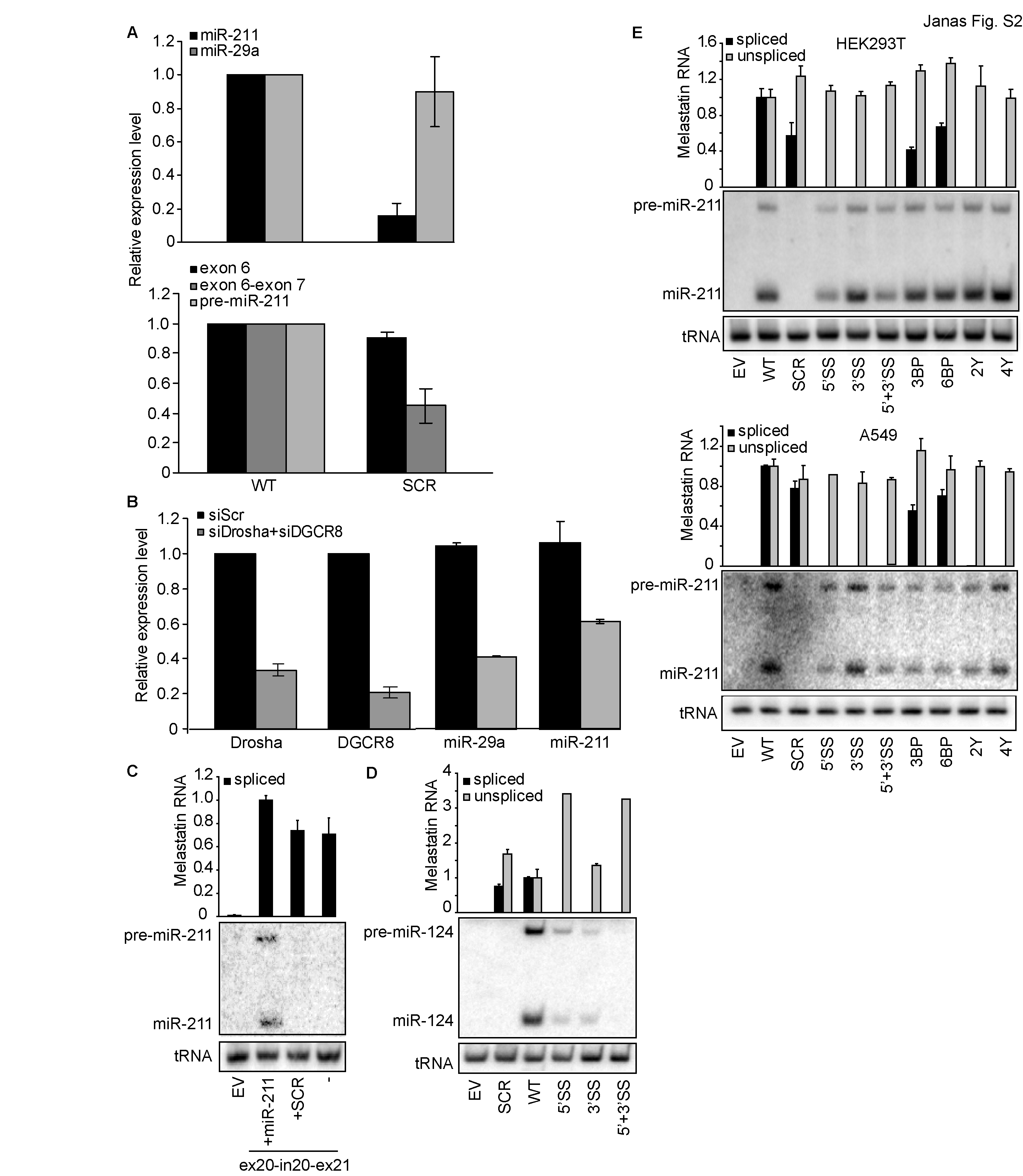

Supplement: Figure S2 — Cooperativity between splicing and microprocessing is miRNA- and intronic context-independent. (A) Replacing miR-211 with a SCR sequence in the melastatin mini-gene decreases miR-211 expression and exon 6-exon 7 splicing, but not steady-state mini-gene levels. WT or SCR melastatin mini-gene were transfected into HeLa cells, and the levels of miRNAs (intronic miR-211 and intergenic miR-29a) and mini-gene transcripts (using primers that specifically amplify exon 6, exon 6-exon 7, and pre-miR-211) were assessed by qRT-PCR. (B) Knockdown efficiencies of Drosha and DGCR8 in HeLa cells were assessed by qRT-PCR and normalized to Actin. The functionality of knockdowns was confirmed by qRT-PCR for miR-29a and miR-211. (C) miR-211 microprocessing promotes splicing in an intron-independent manner. miR-211 or a SCR sequence were cloned intro a second melastatin mini-gene containing entire exon 20, entire intron 20, and entire exon 21. HeLa cells were transfected with an empty vector (EV), miR-211-containing mini-gene (+miR-211), SCR-containing mini-gene (+SCR), or completely endogenous mini-gene (−). miR-211 expression was assessed by Northern blotting normalized to tRNA, and exon 20-exon 21 splicing was assessed by qRT-PCR normalized to neomycin. (D) Positive effects of 5′SS recognition on microprocessing and microprocessing on splicing are miRNA-independent. miR-211 in the exon 6-intron 6-exon 7 melastatin mini-gene was replaced by miR-124, and the effects of miR-124 microprocessing on splicing and splicing on miR-124 microprocessing were assessed after transfection of WT and mutant mini-genes into HeLa cells. Spliced exon 6-exon 7 junctions and unspliced exon 6-intron 6 junctions were assessed by qRT-PCR normalized to neomycin, and miR-124 expression was assessed by Northern blotting normalized to tRNA. (E) Positive effects of 5′SS recognition on microprocessing and microprocessing on splicing are cell type-independent. Either empty vector (EV) or vectors containing WT or mu [file pgen.1002330.s002.tif]

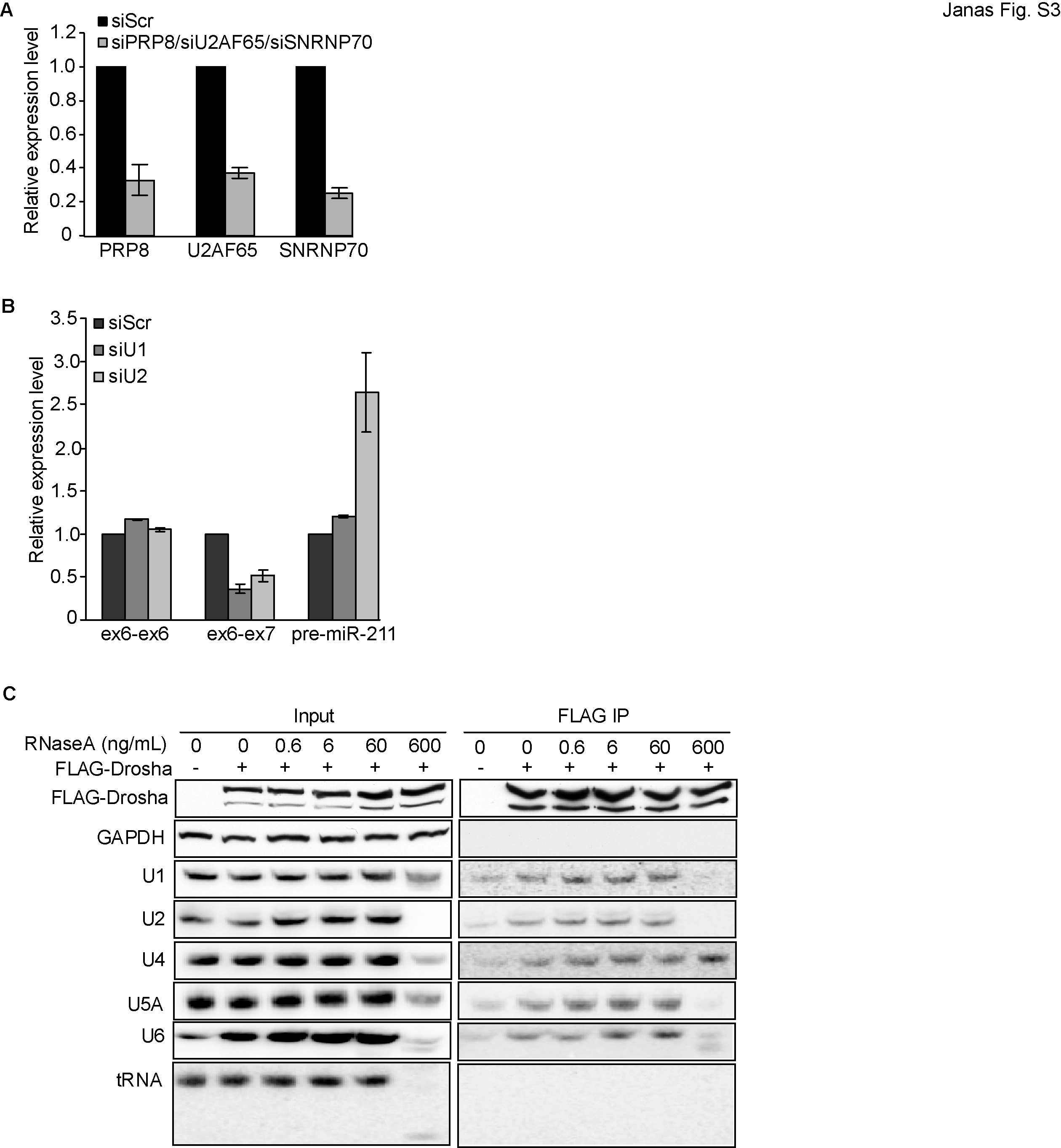

Supplement: Figure S3 — The spliceosome and the Microprocessor are mechanistically and physically coupled. (A) Knockdown efficiencies of indicated splicing factors (PRP8, U2AF65, and SNRNP70) in HeLa cells were assessed by qRT-PCR and normalized to Actin. (B) Knockdown of U1 (SNRPNP70+PRP8) and U2 (U2AF65+PRP8) splicing factors decreases exon 6-exon 7 splicing but not mini-gene transcript steady-state levels. Scr or splicing factor-specific siRNAs were transfected into HeLa cells, and the levels of mini-gene transcripts (using primers that specifically amplify exon 6, exon 6-exon 7, and pre-miR-211) were assessed by qRT-PCR. (C) The interaction between Drosha and the spliceosome is RNA-independent. Empty (−) or FLAG-Drosha-expressing (+) vectors were transfected into HeLa cells, and FLAG-Drosha was immunoprecipitated with anti-FLAG beads in the absence or presence of increasing concentrations of RNaseA. Inputs and anti-FLAG-Drosha immunoprecipitates were analyzed for proteins (FLAG-Drosha and GAPDH) and RNAs (U1, U2, U4, U5A, U6, and tRNA) by Western and Northern blotting, respectively. (TIF) [file pgen.1002330.s003.tif]

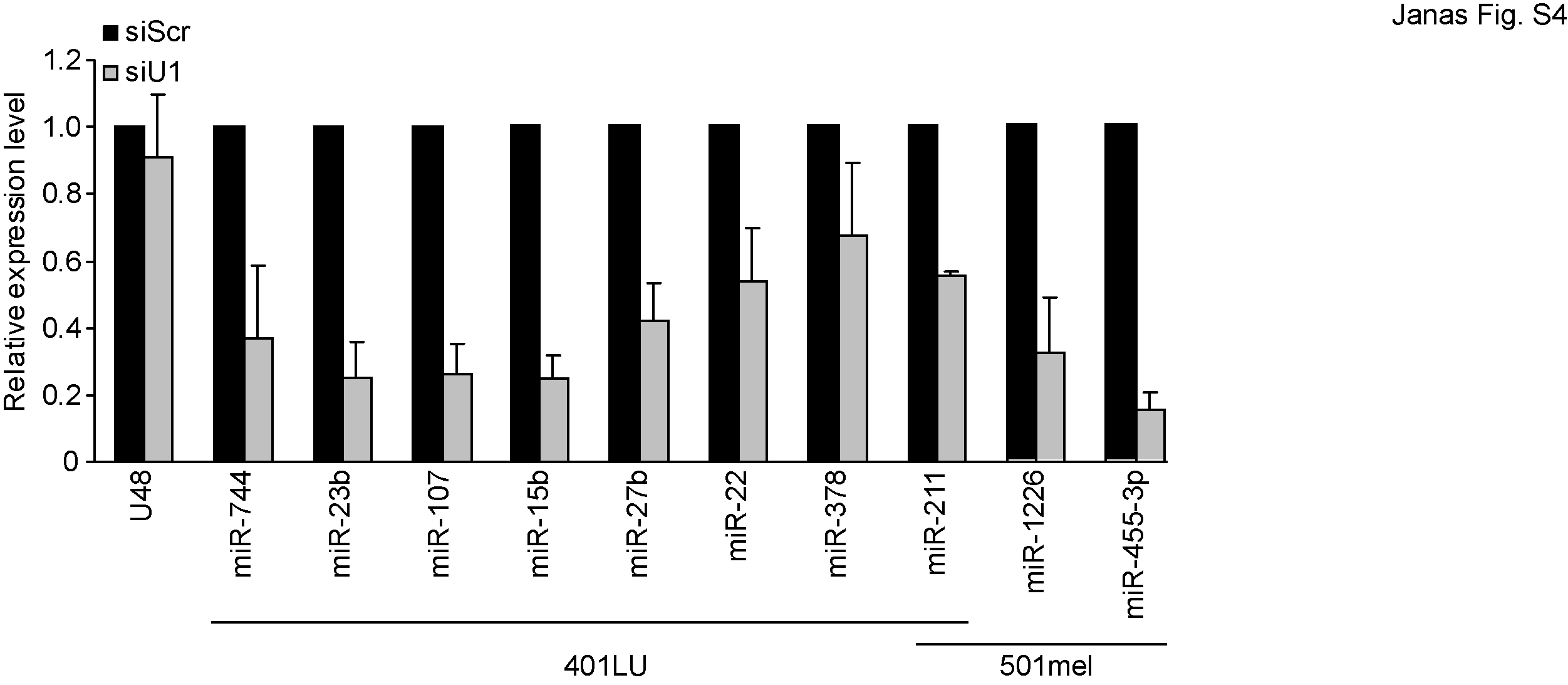

Supplement: Figure S4 — Validation of miRNA expression profiling data. Intronic miRNAs that decreased more than two-fold after U1 (SNRNP70+PRP8) knockdown as assessed by the microarray were validated by qRT-PCR and normalized to U48 in the indicated melanoma cell lines. (TIF) [file pgen.1002330.s004.tif]
