# Supplementary material for: Feed-Forward Microprocessing and Splicing Activities at a MicroRNA–Containing Intron
Source: PLoS Genet. 2011 Oct 20;7(10):e1002330. doi: 10.1371/journal.pgen.1002330 (PMC3197686; doi:10.1371/journal.pgen.1002330)
Supplement: Table S1 — Primers used in these studies. (DOC) [file pgen.1002330.s005.doc]

Janas Table S1

| hDrosha F | TCAGGTGGGAGATTCTAACGTGGT |
| --- | --- |
| hDrosha R | TCATCGAGCTTCGTCTTTGGAGGT |
| hSNRP70 | ID: Hs01094449* |
| hU2AF65 | ID: Hs00200737* |
| hPRPF8 | ID: Hs01556851* |
| hTRPM1ex4-ex5F | ATGCAGCCCAAGCTGAAACAAGTC |
| hTRPM1ex4-ex5R | TGGAGGAGTGGTCTTTCAAGGCAT |
| hTRPM1ex6-ex7F | GCACATCTCCCTGCAGAAGATCAA |
| hTRPM1ex6-ex7R | TCCAAGACGATGGACACCACGTTA |
| hTRPM1ex16-ex17F | GCAGTGCTGATGAAACGCCAGAAA |
| hTRPM1ex16-ex17R | AATAACTCCAAAGCAAGCTGGCCG |
| hTRPM1ex19-ex20F | GCAAATGCAGATGCTGGCTCAAGA |
| hTRPM1ex19-ex20R | AGCAGCAGGTAGCCCAAGTATGAT |
| pre-miR211 F | TGTGGGCTTCCCTTTGTCATCCTT |
| pre-miR211 R | TGCTGTGGGAAGTGACAACTGA |
| hTRPM1ex6-int6F | ACAATGGCACCCTGGGCAAGTAT |
| hTRPM1ex6-int6R | CCTTCTCAGCCTTGTTTCCACTGT |
| hTRPM1int6-ex7F | GCGAATGTGGTTTGTGTTGCAGGA |
| hTRPM1int6-ex7R | TCACAAATCACCACAGGGATGGGA |
| hTRPM1ex6-ex6F | TCTCTGTGCTCAACAACTCCCACA |
| hTRPM1ex6-ex6R | TGATCTTCTGCAGGGAGATGTGCT |
| hActinF | ATTGCCGACAGGATGCAGAA |
| hActinR | GCTGATCCACATCTGCTGGAA |
| BAC clone RP11-348B17 F | GCCAAGCTTATAATGTCCAACCCTCTAAGTAAGCTCTCTGTG |
| BAC clone RP11-348B17 R | GCGGATCCTCACCACAGGGATGGGAGGCTCTTCTTGCAGG |
| 5’SS mut F | GAAGATCAACACAACAAAGTGCTGGCAAAGGCA |
| 5’SS mut R | TGCCTTTGCCAGCACTTTGTTGTGTTGATCTTCTGC |
| 3’SS mut F | GGCGAATGTGGTTTGTGTTGCCAGACTGGGGC |
| 3’SS mut R | GCAACACAAACCACATTCGCCCATACCACCTG |
| SCR mut F | GGCTTCCCTTTGTCATCCTTCGTCCCACAGCACGGAGCTTCG |
| SCR mut R | AGGATGACAAAGGGAAGCCCACAAGTCTGCCCTGCTCAGAGCCCTAGG |
| 3BP mut F | AGTGCAGGGCACAGGTGCTTTGCGCGAATGTGGTTTGTGT |
| 3BP mut R | ACACAAACCACATTCGCGCAAAGCACCTGTGCCCTGCACT |
| 6BP mut F | GGGCACAGGTGCTTTGCCCGTTTGTGGTTTGTGTTGCAGG |
| 6BP mut R | CCTGCAACACAAACCACAAACGGGCAAAGCACCTGTGCCC |
| 2Y mut F | GGTGGTATGGGCGAATGAGGTGTGTGTTGCAGGACTGGGG |
| 2Y mut R | CCCCAGTCCTGCAACACACACCTCATTCGCCCATACCACC |
| 4Y mut F | GGTATGGGCGAATGAGGTGTGAGGTGCAGGACTGGGGCAGG |
| 4Y mut R | CCTGCCCCAGTCCTGCACCTCACACCTCATTCGCCCATACC |
| Drosha siRNA mut F | GAAGGACACTTAACTTTATTACGTAGCTCTTTGGTGAAT |
| Drosha siRNA mut R | ATTCACCAAAGAGCTACGTAATAAAGTTAAGTGTCCTTC |
| Drosha E1045Q mut F | GGCCAATTGTTTTCAAGCGTTAATAGGAGC |
| Drosha E1045Q mut R | GCTCCTATTAACGCTTGAAAACAATTGGCC |
| Drosha E1222Q mut F | GGCGGACCTTTTGCAATCATTTATTGCAGC |
| Drosha E1222Q mut R | GCTGCAATAAATGATTGCAAAAGGTCCGCC |
| hTRPM1 ex20-in20 F | ccaggagtggatcgtcatct |
| hTRPM1 ex20-in20 R | ggctggtttcaaattcctga |
| hTRPM1 ex20-ex21 F | tacttgggctacctgctgct |
| hTRPM1 ex20-ex21 R | ccatgtagggctggttctgt |
| Age1-SCR-EcoR1 F | CcggtTCACCTGGCCATGTGACTTGTGGGCTTCCCTTTGTCATCCTTCGCCTAGGGCTCTGAGCAGGGCAGACTTGTGGGCTTCCCTTTGTCATCCTTCGTCCCACAGCACGGAGg |
| Age1-SCR-EcoR1 F | AattcCTCCGTGCTGTGGGACGAAGGATGACAAAGGGAAGCCCACAAGTCTGCCCTGCTCAGAGCCCTAGGCGAAGGATGACAAAGGGAAGCCCACAAGTCACATGGCCAGGTGAa |
| InsertAgeIF | ggagtctgtctcaccggtgggccccacctcaa |
| InsertAgeIR | ttgaggtggggcccaccggtgagacagactcc |
| InsertEcorIF | ctttgggccccacctgaattcgtcattggtacccac |
| InsertEcoRIR | gtgggtaccaatgacgaattcaggtggggcccaaag |
| Clone minigene2HindIIIF | cccaagcttataatgtccaaccctctaagtaagctctctgtgatatcatacttgggctacctgctgctgtttaactacgtcatcctggtg |
| Clone minigene2NotIR | ATAGTTTAGCGGCCGCTCATCTTTCCAATCATCATCACGTATGGCCCCAG |
| PCR minigene2 F | atatcatacttgggctacctgctgctgtttaac |
| PCR minigene2 R | CATCTTTCCAATCATCATCACGTATGGCCCC |
| TRPM1introAgeI F | acttgtaagtttttacaacaccggttttcacctggccatgtgacttg |
| TRPM1introAgeI R | caagtcacatggccaggtgaaaaccggtgttgtaaaaacttacaagt |
| TRPM1introPmlI F | cacttcccacagcacgtggcttcgaggactccag |
| TRPM1introPmlI R | ctggagtcctcgaagccacgtgctgtgggaagtg |
| TRPM1introSacII F | gctcagttgtcacttcccacaccgcggagcttcga |
| TRPM1introSacII R | tcgaagctccgcggtgtgggaagtgacaactga |
| AgeI-PremiR124-SacII F | ccggtAGGCCTCTCTCTCCGTGTTCACAGCGGACCTTGATTTAAATGTCCATACAATTAAGGCACGCGGTGAATGCCAAGAATGGGGCTGccgc |
| AgeI-PremiR124-SacII R | ggCAGCCCCATTCTTGGCATTCACCGCGTGCCTTAATTGTATGGACATTTAAATCAAGGTCCGCTGTGAACACGGAGAGAGAGGCCTa |
| AgeI Pre-miR-124 Pml1 F | ccggtAGGCCTCTCTCTCCGTGTTCACAGCGGACCTTGATTTAAATGTCCATACAATTAAGGCACGCGGTGAATGCCAAGAATGGGGCTGcac |
| AgeI Pre-miR-124 Pml1 R | gtgCAGCCCCATTCTTGGCATTCACCGCGTGCCTTAATTGTATGGACATTTAAATCAAGGTCCGCTGTGAACACGGAGAGAGAGGCCTa |

* ABI gene expression assay
