# Supplementary material for: Feed-Forward Microprocessing and Splicing Activities at a MicroRNA–Containing Intron
Source: PLoS Genet. 2011 Oct 20;7(10):e1002330. doi: 10.1371/journal.pgen.1002330 (PMC3197686; doi:10.1371/journal.pgen.1002330)
Supplement: Table S2 — Normalized miRNA expression levels (Ct value threshold ≤30) after U1 (SNRNP70+PRP8) knockdown in melanoma cell lines 451LU and 501mel as detected by the microarray. (DOC) [file pgen.1002330.s006.doc]

Janas Table S1

| **miRNA name** | **miRNA type** | **451LU: log_2_(siU1/siScr)** | **501mel: log_2_(siU1/siScr)** |
| --- | --- | --- | --- |
| hsa-miR-23b-4373073 | intronic | -2.461347369 |  |
| hsa-miR-744#-002325 | intronic | -2.246399636 | -1.135980315 |
| hsa-miR-16-1#-002420 | intronic | -1.890619636 | 1.030761685 |
| hsa-miR-15b#-002173 | intronic | -1.711558636 | 0.742494685 |
| hsa-miR-27b#-002174 | intronic | -1.673878636 |  |
| hsa-miR-1233-002768 | intronic | -1.470377636 | -0.475986315 |
| hsa-miR-22-4373079 | intronic | -1.393745369 |  |
| hsa-miR-22#-002301 | intronic | -1.328154636 | -0.163119315 |
| hsa-miR-378-000567 | intronic | -1.292740636 |  |
| hsa-miR-378-002243 | intronic | -1.241810636 | 0.720382685 |
| hsa-miR-361-5p-4373035 | intronic | -1.158529369 | -0.456200621 |
| hsa-miR-491-5p-4381053 | intronic | -1.103907369 | -0.494433621 |
| hsa-miR-590-3P-002677 | intronic | -1.098030636 | 0.651800685 |
| hsa-miR-107-4373154 | intronic | -1.050340369 | -0.122014621 |
| hsa-miR-98-4373009 | intronic | -0.885245369 | 0.982537379 |
| hsa-miR-597-4380960 | intronic | -0.853671369 | 0.473272379 |
| hsa-miR-140-3p-4395345 | intronic | -0.823941369 | -0.223252621 |
| hsa-miR-941-002183 | intronic | -0.818209636 | 0.918850685 |
| hsa-miR-10b#-002315 | intronic | -0.807716636 | 0.597156685 |
| hsa-miR-590-5p-4395176 | intronic | -0.766005369 | 0.810960379 |
| hsa-miR-340-4395369 | intronic | -0.747885369 | 0.337980379 |
| hsa-miR-454-4395434 | intronic | -0.717774369 | 0.438486379 |
| hsa-miR-186-4395396 | intronic | -0.693716369 | 0.124585379 |
| hsa-miR-149-4395366 | intronic | -0.666993369 | 0.314355379 |
| hsa-miR-218-4373081 | intronic | -0.661992369 |  |
| hsa-miR-301a-4373064 | intronic | -0.653949369 | 0.253710379 |
| hsa-miR-148b#-002160 | intronic | -0.625710636 | 0.279770685 |
| hsa-miR-30e-3p-000422 | intronic | -0.613292636 | 0.245152685 |
| hsa-miR-151-3p-002254 | intronic | -0.610108636 | 0.361301685 |
| hsa-miR-152-4395170 | intronic | -0.582435369 | 0.653726379 |
| hsa-miR-577-002675 | intronic | -0.561024636 |  |
| hsa-miR-148b-4373129 | intronic | -0.557982369 | 0.159700379 |
| hsa-miR-628-5p-4395544 | intronic | -0.540161369 | 0.010993379 |
| hsa-miR-151-5P-002642 | intronic | -0.528506636 | 0.074275685 |
| hsa-miR-185-4395382 | intronic | -0.505714369 | -0.214645621 |
| hsa-miR-106b#-002380 | intronic | -0.497572636 | 0.001533685 |
| hsa-miR-9-4373285 | intronic | -0.485136369 | 0.421745379 |
| hsa-miR-942-002187 | intronic | -0.478548636 | 0.287873685 |
| hsa-miR-140-5p-4373374 | intronic | -0.478172369 | -0.114458621 |
| hsa-miR-340#-002259 | intronic | -0.476356636 | 0.151845685 |
| hsa-miR-625#-002432 | intronic | -0.472393636 | 0.153087685 |
| hsa-miR-339-5p-4395368 | intronic | -0.456540369 | -0.417465621 |
| hsa-miR-16-4373121 | intronic | -0.453011369 | 0.116422379 |
| hsa-miR-616-001589 | intronic | -0.451603636 | 0.113212685 |
| hsa-miR-15b-4373122 | intronic | -0.442339369 | -0.129754621 |
| hsa-miR-28-3p-4395557 | intronic | -0.403966369 | -0.163934621 |
| hsa-miR-330-3p-4373047 | intronic | -0.390789369 |  |
| hsa-miR-629-4395547 | intronic | -0.389817369 |  |
| hsa-miR-1249-002868 | intronic | -0.381056636 |  |
| hsa-miR-139-5p-4395400 | intronic | -0.369133369 | -0.149690621 |
| hsa-miR-26b-4395167 | intronic | -0.368064369 | 0.362775379 |
| hsa-miR-1254-002818 | intronic | -0.351511636 | 0.397007685 |
| hsa-miR-652-4395463 | intronic | -0.343761369 | 0.017441379 |
| hsa-miR-425#-002302 | intronic | -0.337030636 | 0.253698685 |
| hsa-miR-601-001558 | intronic | -0.335631636 | -0.236484315 |
| hsa-let-7g-4395393 | intronic | -0.330078369 | 0.241522379 |
| hsa-miR-576-3p-4395462 | intronic | -0.326809369 | 0.453451379 |
| hsa-miR-106b-4373155 | intronic | -0.318795369 | 0.651663379 |
| hsa-miR-1180-002847 | intronic | -0.301903636 | -0.205930315 |
| hsa-miR-204-4373094 | intronic | -0.299982369 |  |
| hsa-miR-625-4395542 | intronic | -0.296453369 | 0.245768379 |
| hsa-miR-1260-002896 | intronic | -0.286185636 | 0.374304685 |
| hsa-miR-338-5P-002658 | intronic | -0.277281636 | 0.735996685 |
| hsa-miR-455-3p-4395355 | intronic | -0.273589369 | -2.169027621 |
| hsa-miR-744-4395435 | intronic | -0.271326369 | -0.426243621 |
| hsa-miR-211-4373088 | intronic | -0.246501369 | -0.341186621 |
| hsa-miR-126-4395339 | intronic | -0.240960369 | 0.252108379 |
| hsa-miR-455-5p-4378098 | intronic | -0.193673369 |  |
| hsa-miR-589-001543 | intronic | -0.193110636 | 0.172236685 |
| hsa-miR-26b#-002444 | intronic | -0.189304636 |  |
| hsa-miR-1274A-002883 | intronic | -0.174092636 | 0.374922685 |
| hsa-miR-25-4373071 | intronic | -0.156583369 | 0.246179379 |
| hsa-miR-181a-2#-002317 | intronic | -0.151312636 | 0.669621685 |
| hsa-miR-30c-4373060 | intronic | -0.150707369 | -0.197223621 |
| hsa-miR-574-3p-4395460 | intronic | -0.134312369 | -0.454937621 |
| hsa-miR-27b-4373068 | intronic | -0.131201369 | 0.090753379 |
| hsa-miR-28-5p-4373067 | intronic | -0.123508369 | 0.084921379 |
| hsa-miR-663B-002857 | intronic | -0.117170636 | 0.600761685 |
| hsa-miR-423-5p-4395451 | intronic | -0.117164369 | -0.260373621 |
| hsa-miR-766-001986 | intronic | -0.116388636 | 0.252312685 |
| hsa-miR-425-4380926 | intronic | -0.114631369 | -0.710081621 |
| hsa-miR-26a-4395166 | intronic | -0.100642369 | 0.109513379 |
| hsa-miR-99a-4373008 | intronic | -0.096040369 | -0.986538621 |
| hsa-miR-636-4395199 | intronic | -0.056988369 |  |
| hsa-miR-1274B-002884 | intronic | -0.048890636 | 0.064672685 |
| hsa-let-7c-4373167 | intronic | -0.047477369 | 0.249042379 |
| hsa-miR-454#-001996 | intronic | -0.041197636 | 0.824020685 |
| hsa-miR-342-3p-4395371 | intronic | -0.033725369 | -0.178830621 |
| hsa-miR-642-4380995 | intronic | -0.029709369 | -0.189596621 |
| hsa-miR-1290-002863 | intronic | -0.029252636 | 0.751575685 |
| hsa-miR-128-4395327 | intronic | -0.028909369 | -0.320403621 |
| hsa-miR-339-3p-4395295 | intronic | -0.002700369 | 0.135273379 |
| hsa-miR-103-4373158 | intronic | 0.007355631 | 0.098943379 |
| hsa-miR-579-4395509 | intronic | 0.016355631 | 0.392997379 |
| hsa-miR-93#-002139 | intronic | 0.017498364 | 0.097404685 |
| hsa-miR-93-4373302 | intronic | 0.020890631 | -0.074898621 |
| hsa-miR-598-4395179 | intronic | 0.105328631 |  |
| hsa-miR-671-3p-4395433 | intronic | 0.221756631 | -0.690275621 |
| hsa-miR-629-001562 | intronic | 0.237792364 | 0.297186685 |
| hsa-miR-1201-002781 | intronic | 0.509135364 | 0.881170685 |
| hsa-miR-489-4395469 | intronic | 1.617075631 | -0.089904621 |
| hsa-miR-1226#-002758 | intronic |  | -1.688040315 |
| hsa-miR-1271-002779 | intronic |  | 0.412730685 |
| hsa-miR-1296-002908 | intronic |  | -1.120685315 |
| hsa-miR-25#-002442 | intronic |  | 0.701287685 |
| hsa-miR-141-4373137 | intergenic | -2.352774369 |  |
| hsa-miR-29a#-002447 | intergenic | -1.623457636 |  |
| hsa-miR-501-5p-4373226 | intergenic | -1.549155369 | 0.624404379 |
| hsa-miR-20a#-002437 | intergenic | -1.434647636 |  |
| hsa-miR-191#-002678 | intergenic | -1.401158636 | -0.668998315 |
| hsa-miR-30d#-002305 | intergenic | -1.276144636 | 0.234751685 |
| hsa-miR-19a-4373099 | intergenic | -1.123399369 | 0.716770379 |
| hsa-miR-509-3-5p-4395266 | intergenic | -1.025084369 |  |
| hsa-miR-29b-4373288 | intergenic | -0.984296369 | 0.462306379 |
| hsa-miR-130b#-002114 | intergenic | -0.949384636 | 0.782733685 |
| hsa-miR-222#-002097 | intergenic | -0.933099636 | 0.569095685 |
| hsa-miR-182-4395445 | intergenic | -0.887466369 |  |
| hsa-miR-190b-002263 | intergenic | -0.770569636 |  |
| hsa-miR-362-3p-4395228 | intergenic | -0.761441369 | 0.963694379 |
| hsa-miR-21-4373090 | intergenic | -0.755000369 | 0.687197379 |
| hsa-miR-135b-4395372 | intergenic | -0.754872369 | 0.161327379 |
| hsa-miR-500-4395539 | intergenic | -0.708671369 | -0.444795621 |
| hsa-miR-30d-000420 | intergenic | -0.598432636 | -0.330520315 |
| hsa-miR-374a-4373028 | intergenic | -0.585964369 | -0.059404621 |
| hsa-miR-15a#-002419 | intergenic | -0.569360636 | -0.790899315 |
| hsa-miR-301b-4395503 | intergenic | -0.544121369 | 0.634759379 |
| hsa-miR-31#-002113 | intergenic | -0.503075636 |  |
| hsa-miR-18a#-002423 | intergenic | -0.486080636 | -0.372605315 |
| hsa-miR-1275-002840 | intergenic | -0.484991636 | -0.427213315 |
| hsa-miR-10b-4395329 | intergenic | -0.483052369 | 0.018919379 |
| hsa-miR-509-5p-4395346 | intergenic | -0.473113369 | 0.512619379 |
| hsa-miR-30a-3p-000416 | intergenic | -0.473053636 | -0.076900315 |
| hsa-miR-27a#-002445 | intergenic | -0.468998636 | 0.188005685 |
| hsa-miR-886-3p-4395305 | intergenic | -0.458709369 |  |
| hsa-miR-34a#-002316 | intergenic | -0.453158636 | 0.687411685 |
| hsa-miR-551b-4380945 | intergenic | -0.449031369 |  |
| hsa-miR-512-3p-4381034 | intergenic | -0.438519369 |  |
| hsa-miR-30a-5p-000417 | intergenic | -0.428965636 | -0.394754315 |
| hsa-miR-424-4373201 | intergenic | -0.420517369 |  |
| hsa-miR-660-4380925 | intergenic | -0.394570369 | 0.745659379 |
| hsa-miR-34a-4395168 | intergenic | -0.394397369 | 0.352678379 |
| hsa-miR-125a-3p-4395310 | intergenic | -0.373349369 | -0.336981621 |
| hsa-miR-31-4395390 | intergenic | -0.348308369 |  |
| hsa-miR-200c-4395411 | intergenic | -0.345223369 |  |
| hsa-miR-191-4395410 | intergenic | -0.340026369 | 0.143290379 |
| hsa-miR-508-3p-4373233 | intergenic | -0.314912369 | 1.550302379 |
| hsa-miR-212-4373087 | intergenic | -0.306078369 |  |
| hsa-miR-148a-4373130 | intergenic | -0.285866369 | -0.159673621 |
| hsa-miR-29a-4395223 | intergenic | -0.284473369 | 0.022349379 |
| hsa-miR-17-4395419 | intergenic | -0.274110369 | -0.039317621 |
| hsa-miR-146a-4373132 | intergenic | -0.272758369 | 0.169764379 |
| hsa-miR-222-4395387 | intergenic | -0.267377369 | -0.477604621 |
| hsa-miR-10a-4373153 | intergenic | -0.266122369 | -0.223131621 |
| hsa-miR-331-3p-4373046 | intergenic | -0.263429369 | -0.441058621 |
| hsa-miR-20b-4373263 | intergenic | -0.262132369 | 0.154465379 |
| hsa-miR-138-4395395 | intergenic | -0.254208369 | 0.297251379 |
| hsa-miR-324-3p-4395272 | intergenic | -0.246283369 | -0.232665621 |
| hsa-miR-106a-4395280 | intergenic | -0.245822369 | 0.389528379 |
| hsa-miR-210-4373089 | intergenic | -0.238981369 | -0.407149621 |
| hsa-miR-18a-4395533 | intergenic | -0.236582369 | 0.065807379 |
| hsa-miR-100-4373160 | intergenic | -0.230438369 | -0.041341621 |
| hsa-miR-484-4381032 | intergenic | -0.229354369 | -0.069904621 |
| hsa-miR-769-5p-001998 | intergenic | -0.219278636 | -0.374147315 |
| hsa-let-7b-4395446 | intergenic | -0.218384369 | 0.131433379 |
| hsa-miR-374b-4381045 | intergenic | -0.218198369 | -0.211193621 |
| hsa-miR-221-4373077 | intergenic | -0.216632369 | -0.412171621 |
| hsa-miR-345-4395297 | intergenic | -0.213632369 | -0.213628621 |
| hsa-miR-184-4373113 | intergenic | -0.211286369 | 0.432218379 |
| hsa-miR-125a-5p-4395309 | intergenic | -0.204208369 | 0.046201379 |
| hsa-miR-27a-4373287 | intergenic | -0.201895369 | -0.094547621 |
| hsa-miR-146b-5p-4373178 | intergenic | -0.187573369 | 0.143526379 |
| hsa-miR-502-3p-4395194 | intergenic | -0.184788369 | 0.183852379 |
| hsa-miR-130b-4373144 | intergenic | -0.181715369 | -0.082332621 |
| hsa-miR-708-4395452 | intergenic | -0.179092369 | -0.380451621 |
| hsa-miR-328-4373049 | intergenic | -0.171991369 | -0.959871621 |
| hsa-let-7d-4395394 | intergenic | -0.159065369 | -0.103438621 |
| hsa-miR-505#-002087 | intergenic | -0.136013636 | 0.383918685 |
| hsa-miR-320a-4395388 | intergenic | -0.135708369 | -0.011103621 |
| hsa-miR-30b-4373290 | intergenic | -0.132699369 | -0.093580621 |
| hsa-miR-195-4373105 | intergenic | -0.122467369 | -0.265542621 |
| hsa-miR-192-4373108 | intergenic | -0.121007369 | 0.322622379 |
| hsa-miR-502-5p-4373227 | intergenic | -0.120080369 | 0.499932379 |
| hsa-miR-362-5p-4378092 | intergenic | -0.105521369 | 0.178199379 |
| hsa-miR-296-5p-4373066 | intergenic | -0.090357369 | 0.652612379 |
| hsa-miR-532-5p-4380928 | intergenic | -0.071677369 | -0.009269621 |
| hsa-miR-20a-4373286 | intergenic | -0.062898369 | 0.314993379 |
| hsa-miR-130a-4373145 | intergenic | -0.054670369 | 0.666025379 |
| hsa-miR-886-5p-4395304 | intergenic | -0.052785369 |  |
| hsa-miR-99b-4373007 | intergenic | -0.047082369 | -0.374437621 |
| hsa-miR-324-5p-4373052 | intergenic | -0.035318369 | -0.402841621 |
| hsa-miR-532-3p-4395466 | intergenic | -0.034408369 | -0.142999621 |
| hsa-miR-193a-5p-4395392 | intergenic | -0.033331369 | 0.079397379 |
| hsa-miR-422a-4395408 | intergenic | -0.030973369 | -0.641017621 |
| hsa-miR-197-4373102 | intergenic | -0.007434369 | -0.547971621 |
| hsa-miR-132-4373143 | intergenic | 0.021803631 | -0.252730621 |
| hsa-miR-720-002895 | intergenic | 0.060424364 | 0.245907685 |
| hsa-miR-375-4373027 | intergenic | 0.066297631 |  |
| hsa-let-7e-4395517 | intergenic | 0.092751631 | 0.244314379 |
| hsa-miR-506-4373231 | intergenic | 0.128989631 |  |
| hsa-miR-145-4395389 | intergenic | 0.151617631 |  |
| hsa-miR-193b-4395478 | intergenic | 0.168923631 | -0.453633621 |
| hsa-miR-510-4395352 | intergenic | 0.366895631 |  |
| hsa-miR-1305-002867 | intergenic | 0.645417364 | -0.355434315 |
| hsa-miR-99b#-002196 | intergenic | 0.744403364 |  |
| hsa-miR-29c-4395171 | intergenic | 1.224405631 | -0.314684621 |
| hsa-miR-1269-002789 | intergenic |  | 0.568782685 |
| hsa-miR-1303-002792 | intergenic |  | -0.366424315 |
| hsa-miR-135b#-002159 | intergenic |  | -0.310558315 |
| hsa-miR-187-4373307 | intergenic |  | 0.561366379 |
| hsa-miR-196b-4395326 | intergenic |  | 0.829563379 |
| hsa-miR-203-4373095 | intergenic |  | 0.888685379 |
| hsa-miR-206-000510 | intergenic |  | -0.510089315 |
| hsa-miR-331-5p-4395344 | intergenic |  | -0.079140621 |
| hsa-miR-34b-002102 | intergenic |  | 0.750072685 |
| hsa-miR-424#-002309 | intergenic |  | -0.054104315 |
