# Supplementary material for: Feed-Forward Microprocessing and Splicing Activities at a MicroRNA–Containing Intron
Source: PLoS Genet. 2011 Oct 20;7(10):e1002330. doi: 10.1371/journal.pgen.1002330 (PMC3197686; doi:10.1371/journal.pgen.1002330)
Supplement: Table S3 — Intronic and intergenic miRNAs that decrease at least two fold after U1 (SNRNP70+PRP8) knockdown in melanoma cell lines 451LU and 501mel as detected by the microarray; #, miRNA* strand; N/A, not detected by the microarray. (DOC) [file pgen.1002330.s007.doc]

|  | **miRNAs with ≥ 2-fold decrease after U1 depletion** | | | **complementary miRNA strand levels** | | | **miRNA decrease in the second cell line** | | |
| --- | --- | --- | --- | --- | --- | --- | --- | --- | --- |
|  | **miRNA name** | **log_2_(siU1/siScr)** | **cell line** | **miRNA name** | **log_2_(siU1/siScr)** | **cell line** | **miRNA name** | **log_2_(siU1/siScr)** | **cell line** |
| **Intronic** | hsa-miR-744#-002325 | -2.246399636 | 451LU | hsa-miR-744-4395435 | -0.271326369 | 451LU | - |  |  |
|  | hsa-miR-744#-002325 | -1.135980315 | 501mel | hsa-miR-744-4395435 | -0.426243621 | 501mel | - |  |  |
|  | hsa-miR-590-3P-002677 | -1.098030636 | 451LU | N/A |  |  | hsa-miR-590-3P-002677 | 0.651800685 | 501mel |
|  | hsa-miR-491-5p-4381053 | -1.103907369 | 451LU | N/A |  |  | hsa-miR-491-5p-4381053 | -0.494433621 | 501mel |
|  | hsa-miR-455-3p-4395355 | -2.169027621 | 501mel | N/A |  |  | hsa-miR-455-3p-4395355 | -0.273589369 | 451LU |
|  | hsa-miR-378-002243 | -1.241810636 | 451LU | N/A |  |  | hsa-miR-378-002243 | 0.720382685 | 501mel |
|  | hsa-miR-378-000567 | -1.292740636 | 451LU | N/A |  |  | N/A |  |  |
|  | hsa-miR-361-5p-4373035 | -1.158529369 | 451LU | N/A |  |  | hsa-miR-361-5p-4373035 | -0.456200621 | 501mel |
|  | hsa-miR-27b#-002174 | -1.673878636 | 451LU | hsa-miR-27b-4373068 | -0.131201369 | 451LU | N/A |  |  |
|  | hsa-miR-23b-4373073 | -2.461347369 | 451LU | N/A |  |  | N/A |  |  |
|  | hsa-miR-22-4373079 | -1.393745369 | 451LU | - |  |  | N/A |  |  |
|  | hsa-miR-22#-002301 | -1.328154636 | 451LU | - |  |  | hsa-miR-22#-002301 | -0.163119315 | 501mel |
|  | hsa-miR-16-1#-002420 | -1.890619636 | 451LU | N/A |  |  | hsa-miR-16-1#-002420 | 1.030761685 | 501mel |
|  | hsa-miR-15b#-002173 | -1.711558636 | 451LU | hsa-miR-15b-4373122 | -0.442339369 | 451LU | hsa-miR-15b#-002173 | 0.742494685 | 501mel |
|  | hsa-miR-1296-002908 | -1.120685315 | 501mel | N/A |  |  | N/A |  |  |
|  | hsa-miR-1233-002768 | -1.470377636 | 451LU | N/A |  |  | hsa-miR-1233-002768 | -0.475986315 | 501mel |
|  | hsa-miR-1226#-002758 | -1.688040315 | 501mel | N/A |  |  | N/A |  |  |
|  | hsa-miR-107-4373154 | -1.050340369 | 451LU | N/A |  |  | hsa-miR-107-4373154 | -0.122014621 | 501mel |
| **Intergenic** | hsa-miR-509-3-5p-4395266 | -1.025084369 | 451LU | N/A |  |  | N/A |  |  |
|  | hsa-miR-501-5p-4373226 | -1.549155369 | 451LU | N/A |  |  | hsa-miR-501-5p-4373226 | 0.624404379 | 501mel |
|  | hsa-miR-30d#-002305 | -1.276144636 | 451LU | hsa-miR-30d-000420 | -0.598432636 | 451LU | hsa-miR-30d#-002305 | 0.234751685 | 501mel |
|  | hsa-miR-29a#-002447 | -1.623457636 | 451LU | hsa-miR-29a-4395223 | -0.284473369 | 451LU | N/A |  |  |
|  | hsa-miR-20a#-002437 | -1.434647636 | 451LU | hsa-miR-20a-4373286 | -0.062898369 | 451LU | N/A |  |  |
|  | hsa-miR-19a-4373099 | -1.123399369 | 451LU | N/A |  |  | hsa-miR-19a-4373099 | 0.716770379 | 501mel |
|  | hsa-miR-191#-002678 | -1.401158636 | 451LU | hsa-miR-191-4395410 | -0.340026369 | 451LU | hsa-miR-191#-002678 | -0.668998315 | 501mel |
|  | hsa-miR-141-4373137 | -2.352774369 | 451LU | N/A |  |  | N/A |  |  |

Janas Table S3
